# X-ray Tomography Applied to Electrochemical Devices and Electrocatalysis

**DOI:** 10.1021/acs.chemrev.2c00873

**Published:** 2023-08-14

**Authors:** Jack T. Lang, Devashish Kulkarni, Collin W. Foster, Ying Huang, Mitchell A. Sepe, Sirivatch Shimpalee, Dilworth Y. Parkinson, Iryna V. Zenyuk

**Affiliations:** † Department of Chemical and Biomolecular Engineering, University of California, Irvine, California 92617, United States; ‡ National Fuel Cell Research Center, University of California, Irvine, California 92617, United States; § Department of Materials Science and Engineering, University of California, Irvine, California 92617, United States; ∥ Department of Aerospace Engineering, University of Illinois at Urbana−Champaign, Urbana, Illinois 61820, United States; ⊥ Hydrogen and Fuel Cell Center, Department of Chemical Engineering, University of South Carolina, Columbia, South Carolina 29208, United States; # Advanced Light Source, Lawrence Berkeley National Laboratory, Berkeley, California 94720, United States

## Abstract

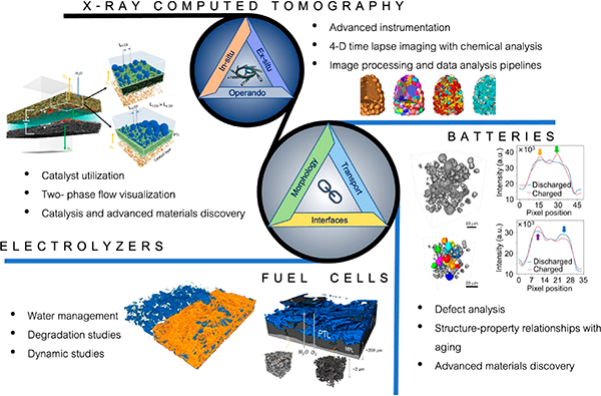

X-ray computed tomography (CT) is a nondestructive three-dimensional (3D) imaging technique used for studying morphological properties of porous and nonporous materials. In the field of electrocatalysis, X-ray CT is mainly used to quantify the morphology of electrodes and extract information such as porosity, tortuosity, pore-size distribution, and other relevant properties. For electrochemical systems such as fuel cells, electrolyzers, and redox flow batteries, X-ray CT gives the ability to study evolution of critical features of interest in ex situ, in situ, and operando environments. These include catalyst degradation, interface evolution under real conditions, formation of new phases (water and oxygen), and dynamics of transport processes. These studies enable more efficient device and electrode designs that will ultimately contribute to widespread decarbonization efforts.

## 1 Introduction

### 1.1 Overview

The global movement toward positive climate action by widespread decarbonization efforts have spurred the growth and adoption of renewable energy power generation. Consequently, the market for electrochemical energy conversion devices has increased significantly and future projections have shown highly optimistic trends for market penetration.^1−3^ Technologies such as fuel cells and batteries have already been used commercially for transportation applications and have proven to be extremely versatile and reliable. Additionally, electrochemical power-to-gas conversion technologies, like electrolyzers for hydrogen production and carbon dioxide reduction, are paving new pathways to offset fossil fuel use in industrial feedstocks in hard to decarbonize sectors like metal refining, chemical manufacturing, and aviation.^4−6^ Reducing costs and increasing the efficiencies of these technologies are important for meeting policy targets and to enable faster commercialization. In general, these technologies often use porous media and precious metal catalysts for multiscale species transport and to facilitate electrochemical reactions. Understanding the underlying mechanisms of electrocatalysis, transport phenomena, and failure mechanisms is critical for designing vital components and interfaces for electrochemical devices.^7^ Hence, micro- and nano-scale imaging techniques are needed to capture these mechanisms with minimal system perturbations.

X-ray CT is a nondestructive 3D technique that was initially developed for medical imaging but is now extensively used in materials science and scientific imaging due to radical technical advances in the past 20 years.^8−10^ X-ray CT was first invented around 1970 and was largely used for medical research until the early 2000s. Since then, the number of publications on its use in other fields has increased exponentially. Out of these, general engineering, materials science, chemical engineering, chemistry, and energy are the top five fields thanks to the technical advances in lab scale and synchrotron X-ray instrumentation and data processing. With the development of synchrotron X-ray CT beamlines and advanced image processing pipelines, it is now possible to achieve tens of nanometers scale spatial and sub-second temporal resolution to reconstruct 3-D volumes with thousands of images collected with a 180-degree rotation of a sample on the stage.^11^

**Figure 1:**
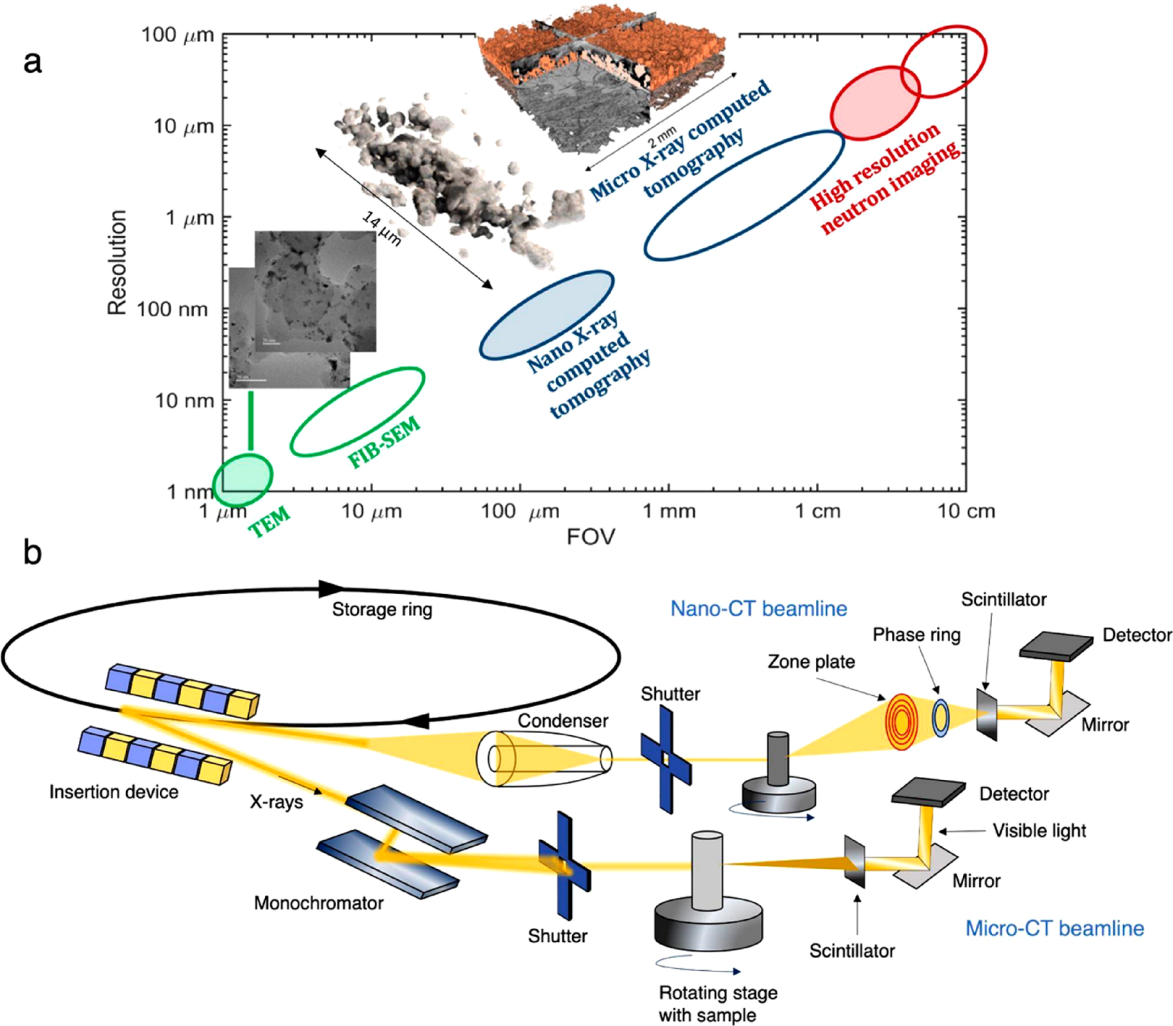
(a) Spatial resolution against field of view (FOV) for various imaging techniques relevant for studying electrochemical devices. Adapted with permission from ref (12). Copyright 2020 IOP Publishing under Creative Commons Attribution 4.0 license (https://creativecommons.org/licenses/by/4.0/). (b) Schematic of typical synchrotron micro- and nano-CT beamlines showing X-ray generation and optics. The distance between the scintillator and the sample can be varied based on either absorption contrast or phase contrast imaging. Beamline optics like the zone plates and optical lenses can be changed based on imaging needs.

X-ray CT is a versatile tool, as it spans various time and length scales of imaging. High-resolution 3-D imaging is useful for small samples or specified regions of interest (ROIs) from large samples. Low-resolution X-ray CT can be used to study samples of larger sizes or in a high throughput mode. There is always a trade-off between the spatial resolution and the field of view (FOV), since it is necessary to resolve a feature within the acceptable limits of the signal-to-noise ratio.^12^Figure 1a shows different imaging techniques with their spatial resolution plotted against the FOV. Micro- and nano-CT are two main techniques with micro and nanoscale spatial resolutions available for X-ray CT. Micro-CT can have a FOV from 0.1 cm to several centimeters, but nano-CT has a much smaller FOV ranging from tens of micrometers to below 1 mm. Based on the spatial resolution, micro- and nano-CT instruments can have different X-ray optics or detectors. A typical synchrotron micro-CT and nano-CT beamline schematics are shown in Figure 1b. X-rays are guided by a monochromator and mirrors to reach the sample on the rotating stage. After the X-rays pass through the sample, they are collected by the detector. To reach nanoscale resolution, various optical elements are added along the beam path for nano-CT beamline, as shown by Figure 1b. These include a beam-shaping condenser, zone plate, and sometimes a phase ring.

In more advanced modes, X-ray CT can be used to track dynamic physical and chemical phenomena under various operando environments stimulating temperature, pressure, chemical, or electrochemical reactions in a 4-D imaging regime considering time as the fourth dimension.^13,14^ This 4-D time lapse imaging can be used to corroborate multiphysics and mathematical modeling predictions with substantial accuracy. The time resolution of the X-ray CT technique varies depending on the beamline. Currently, the TOMCAT beamline at Paul Scherer Institute, Switzerland can achieve subsecond scans with high accuracy.^15−18^ For operando studies, a synchronization of stage rotation with a slip-ring for tubing and wires needs to be achieved, which is not available on most micro-CT beamlines. Alternatively, millisecond imaging resolution is widely available at synchrotron facilities for radiography studies, where 2D imaging instead of 3D imaging is performed.^19^

Depending on the distance of the sample from the detector or use of optics such as phase rings, X-ray CT can be conducted in absorption or phase contrast mode, depending on the type of information needed for the sample imaged. The absorption contrast mode produces image contrast based on the degree of X-ray absorption by a material and is most suitable when the sample under investigation has two or more phases having different attenuation coefficients that can be distinguished easily during image processing. Phase contrast imaging uses phase separation in diffracted X-ray beams and is suited when the sample has different phases having relatively close attenuation coefficients (densities or thicknesses) and visualizing phase boundaries is of relevance. This method is especially valuable for soft materials, such as carbons or oxides, where the difference between carbon, oxides, other soft materials, and air or other gas will be difficult to detect in absorption mode. Both of these modes can be used in conjunction to circumvent the drawbacks of individual modes and enhance visibility of features of interest. For example, Komini Babu et al.^20^ used the results of the two modes to resolve the active material and the carbon binder phase in a lithium-ion battery cathode.

Furthermore, X-ray CT can also operate with diffraction mode (XRD), fluorescence mode (XRF), and X-ray absorption near edge spectroscopy (XANES) depending on type of instrumentation, thus giving combined information on the spatial morphology and chemical nature of the system under study.^21,22^ XRD provides crystallographic, structural information on the material, as well as presence of specific phases. XRF provides information about the relative amount of different elements present in the sample. XANES provides information about oxidation state, geometry, and electronic configuration of the absorber element. Advances in phase retrieval algorithms make it possible to combine these ptychography techniques with X-ray imaging, thus making it possible to study features much smaller than the spatial resolution.^23,24^

Once a sample has been imaged through X-ray CT, it needs to be processed and analyzed to retrieve the data. This process involves image segmentation, thresholding, and computationally restructuring 2D image slices into 3D structures.^11^ A commonly used program to perform image thresholding for analysis is FIJI or ImageJ.^25^ Important physical properties, such as porosity, pore size distribution, and tortuosity, can be measured using image analysis tools. Fluid flow characteristics, such as fluid permeability, can also be extracted using X-ray CT.

### 1.2 Motivation for Using X-ray CT for Electrocatalysis and Electrochemical Device Studies

X-ray CT is an ideal tool for studying electrochemical devices owing to its versatility and the quality of data obtained.^14^ Here, we first review several competing techniques, focusing on their application in electrocatalysis and electrochemical devices. Many imaging techniques, such as focused ion beam scanning electron microscopy (FIB-SEM), transmission electron microscopy (TEM), and neutron imaging, have been used to study electrochemical devices. However, the quality and type of information obtained with electron techniques vary substantially. As shown by Figure 1a, techniques like SEM and TEM can resolve sub-nanometer features, but the limiting FOV is of the order of tens of nm to 1 μm. In electrochemical devices, these techniques can give insight into the catalyst or electrode morphology, but it is sometimes of interest to observe multi-scale transport phenomenon in operating devices to understand the underlying physics. Furthermore, SEM and TEM techniques have limited in situ environments and mostly do not allow operando environments due to difficulties of sample-holder designs.

With neutron imaging, the neutrons are able to penetrate metals more easily than X-rays and provide high contrast for lighter weight elements which can be helpful for electrochemical in situ studies.^26^ Generally electrochemical devices, such as fuel cells, must be modified very little to be adopted for neutron imaging. Therefore, operando sample holder design is generally not an issue in the field. This imaging technique, however, can be limited by the time of scan and its overall lower resolution than X-rays. Normally, neutron imaging is done in radiography mode because full neutron CT measurements need either hours long exposure times or a resolution that is too low to capture finer details found in the inner layers of the electrochemical cell.^27^

X-ray CT is perhaps the only technique that can be implemented in 3D for real devices (operando), nonintrusively within reasonable time scales. Electron-based techniques have limited depth of penetration and cannot be used on operando devices and are mostly used in idealized in situ environments. As already discussed, neutrons can be competitive with X-rays for studies of water and other H-containing molecules, but their time scales are still prohibitively long for tomography studies. X-ray CT is therefore an ideal tool for operando and time-resolved studies. In the field of electrochemical studies, X-ray CT is used to gather structural and catalyst performance related data with ex situ, in situ, and operando studies.^7,14^ Here we will focus mostly on in situ and operando works, and where they are not available, ex situ studies will be reviewed. In situ refers to the studies that involve conditions of practical catalytic operation, whereas operando studies are those that use in situ conditions for the catalyst while also collecting data on the catalyst performance.^28^ Special cell designs are necessary for operando tests so that the area of interest is sufficiently transparent to X-rays and can be viewed fully by the X-rays from the synchrotron beam. A 4D imaging regime (whether transient study or chemical study) can be used to show how the structure of certain materials is linked to the electrochemical performance.^7,14^ In situ and operando studies are particularly helpful for the electrocatalysts used in fuel cells, electrolyzers, and redox flow batteries. Catalysts are important to study, as many of the limiting factors in electrochemical devices are due to poor reaction kinetics, and understanding electrocatalysts using various in situ techniques can improve device performance and efficiency.

X-ray CT can also investigate transport phenomena like water management in a fuel cell, fuel cell reactivity, and electrode degradation.^29,30^ This review looks into studies of water management in the gas diffusion layers (GDLs) inside fuel cells, as water flooding is one of the major issues for fuel cells operating at low temperatures. Understanding water transport pathways in porous catalyst layers and GDLs can enable better designs of these components. Material degradation during fuel cell operation is one of the most important areas in the field of fuel cells.^31^ Specifically, platinum catalyst dissolution, carbon corrosion, and GDL wettability loss are some of the issues that prevent longevity of fuel cells.^32^ Applying X-ray CT to electrochemical cell studies can help understand cell degradation mechanisms and help mitigate cell degradation and failure through better component design.^14^ As for redox flow batteries, the performance and characteristics of the porous electrodes as well as the effects of fluid flow and electrode liquid saturation are the main focuses for operando studies.^33−35^ Electrodes need to be designed to provide surface area for the reactions but also the necessary porosity for convective and capillary fluid flow. X-ray CT can allow quantification of electrode surface area as well as its morphological properties, and the data can be fed into computational models to extract fluid properties such as permeability. This review details studies related to the fluid flow through porous electrodes and the performance and material characteristics of the electrodes in relevant electrochemical systems.

For water electrolyzers, catalyst layers, interfaces, and the porous transport layer (PTL) morphology can be characterized. One of the challenges for water electrolyzers is reducing precious metal (iridium oxide (IrOx)) loadings without compromising performance and durability. To do so, one must design better interfaces and PTLs. PTLs serve many purposes: water delivery to the catalyst layer, removal of product gas, and thermal and electronic transport.^36^ X-ray CT can provide a view of the inner structure of a PTL that can then be analyzed to give quantitative data to characterize the PTL structure and correlate it to the oxygen distribution from the operando data. Porosity, pore size distribution, and tortuosity are morphological quantities that control mass transport properties and performance at high current densities for a PTL. Electrolyzers can also be used to carry out carbon dioxide (CO_2_) reduction reactions. For the CO_2_ reduction electrolyzers, the gas diffusion electrodes (GDEs) are integral to having the gaseous CO_2_ to be able to flow to the catalyst layer to react.^37^ GDEs allow for more catalyst stability and a triple phase interface between electrolyte, catalyst layer, and product gas.^37^ X-ray CT can allow researchers to view and track catalyst particles, ionomers, and water transport inside the GDEs.^38^ Accelerated stress tests and operando studies can be applied with X-ray CT used along the testing protocol to measure GDE degradation mechanisms as well.^38^ These techniques can help improve electrode design and the CO_2_ reduction electrolyzer performance.

This review will first provide an overview of X-ray CT techniques and associated X-ray CT setups, optics, and outline physics of X-ray transmission and absorption, as well as phase contrast. Image processing and data analysis are critical parts of the technique, and we provide an overview of common methodologies for 3D image reconstruction, visualization, and data quantification. Micro-CT is more broadly available in laboratories and at synchrotrons, whereas nano-CT techniques are more rare. Here we provide an overview of several X-ray nano-CT beamlines and an overview of their capabilities. Next, the review focuses on in situ studies and progresses toward operando studies. Specific technologies that are reviewed in this article include polymer electrolyte fuel cells, proton exchange membrane water electrolyzers, CO_2_ reduction reaction electrolyzers, and redox flow batteries. Lastly, we briefly discuss how the data from X-ray CT can be used for multiscale computational models and conclude the review with an outlook and perspective.

## 2 Techniques Available

### 2.1 Overview of Micro- and Nano-CT, Optics, Phase vs Absorption

The complex refractive index of a material, *n*, describes how both the phase and the intensity of light will be affected when passing through it:
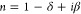
1The imaginary component β describes the attenuation (absorption), and the real component δ describes the phase shift (refraction). Both components contribute to the resulting X-ray images, but depending on the material and the instrument settings, either attenuation or phase contrast might have the dominant contribution to the image.

#### 2.1.1 Attenuation Contrast

**Figure 2:**
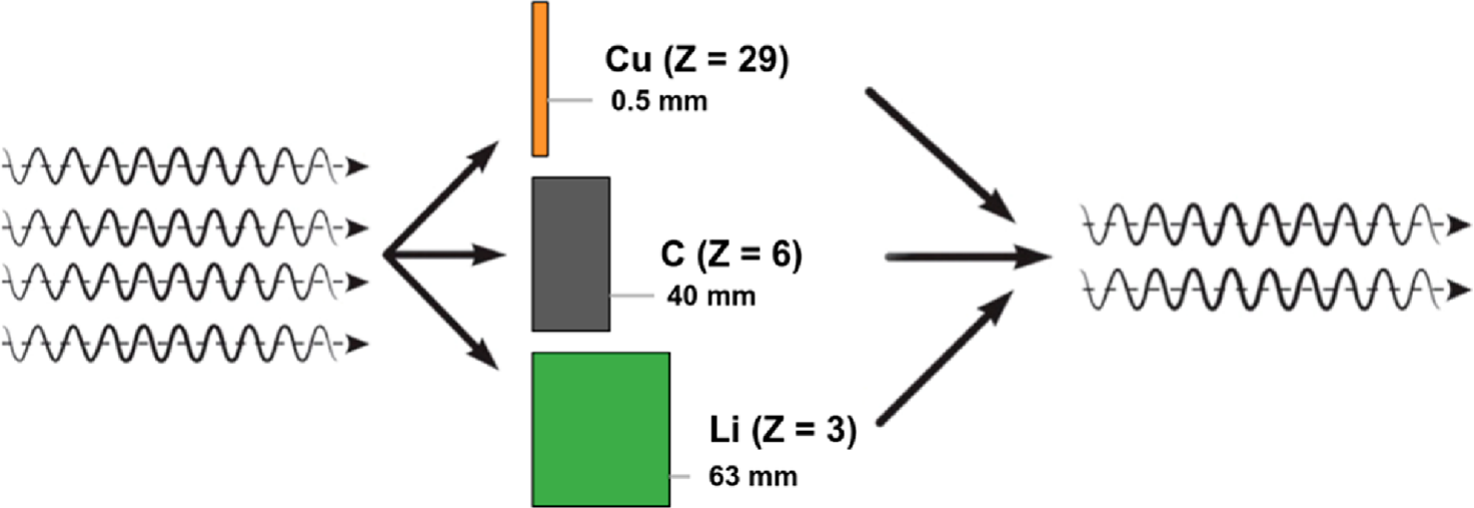
An example of equivalent resulting intensity *I*, if the beam passes through 0.5 mm of Cu, 40 mm of C, or 63 mm of Li. Reprinted with permission from ref (39). Copyright 2015 American Roentgen Ray Society.

For absorption contrast imaging, the intensity measured at each pixel of the image is described by the line integral of the attenuation from the materials through which the beam passes, according to the Beer–Lambert law:
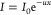
2where *I*_0_ is the incident intensity of light, *u* is the linear attenuation coefficient (which is related to the imaginary part, β, of the index of refraction), and *x* is the path length through a given material. Because the attenuation depends on both the linear attenuation coefficient and the path length, a 2D image at a single wavelength is often insufficient to distinguish materials. Figure 2 shows an example of the equivalent intensity of the beam that passes through three materials of different densities and thicknesses; in each of the cases, the same attenuation is measured. Because Li is the lightest (*Z* = 3), the X-ray intensity from 63 mm of Li will be similar to that of 0.5 mm of Cu. However, because the attenuation coefficient of materials is wavelength-dependent and varies differently for each material, a series of 2D images at a series of different X-ray wavelengths could be used to identify and distinguish materials more fully. This use of imaging over multiple wavelengths is often referred to as X-ray absorption near edge structure (XANES) imaging, which can sometimes differentiate chemical states as well as different materials. The equivalent intensity problem shown in Figure 2 is also a major motivation for collecting tomography data rather than only 2D data—tomographic reconstruction allows quantitative reconstruction of X-ray linear absorption coefficients within a 3D volume.

#### 2.1.2 Phase Contrast

**Figure 3:**
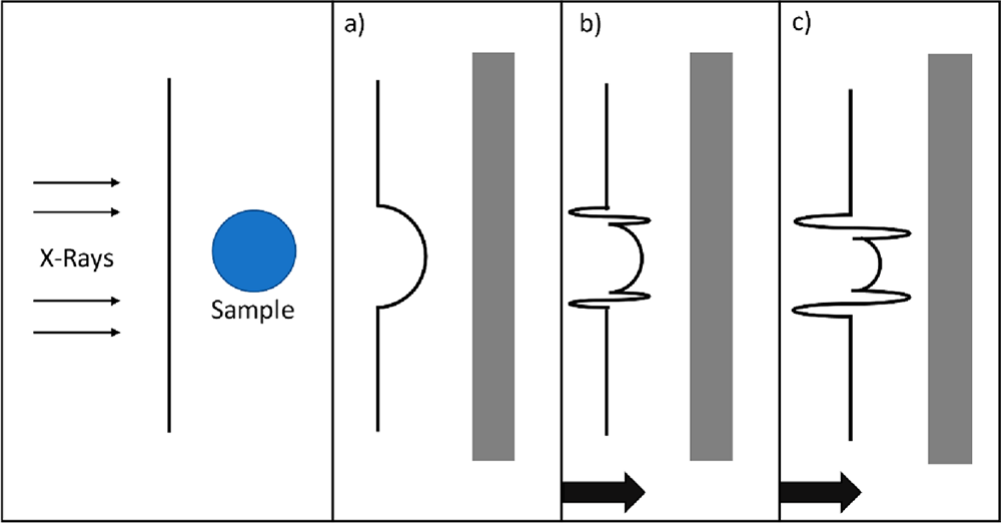
An example of a propagation-based imaging, where depending on the detector position (distance away from the sample imaged), more pronounced phase contrast can be achieved. The arrows in panels b and c indicate the detector’s position is moving from the initial position in panel a.

Although the detectors used for X-ray imaging do not directly measure the beam’s phase shift, phase contrast images can be acquired by transforming the phase shifts generated by the sample into variations in intensity that can be recorded. Propagation-based imaging is the simplest and most common approach to phase contrast imaging. In this mode, the distance from the sample to the detector is increased (and in some cases varied), which allows the radiation refracted by the sample to interfere with the unchanged beam. It is common to use this method with a propagation distance set such that the most obvious effect of the phase contrast is edge enhancement, where structural boundaries of the sample are highlighted, as shown by Figure 3. Propagation-based phase contrast can only be achieved with X-ray sources that have high lateral coherence, which in general is only available at synchrotron sources which have bright X-ray beams (with small source size and high angular collimation).

**Figure 4:**
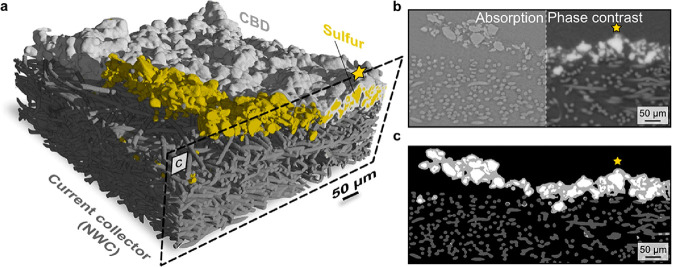
(a) 3D view of a sulfur electrode on NWC. The carbon-binder domain (CBD) is only partially present to show the sulfur phase distribution. (b) X-ray tomogram showing absorption contrast (left side) versus phase contrast (right side). (c) Segmented image. Sulfur is depicted in white, the CBD in light gray, and the fibers of the NWC in dark gray. Reprinted with permission from ref (40). Copyright 2015 Springer Nature.

Phase-contrast imaging is commonly used in the electrochemical community, as many of the imaged materials are soft and cannot be distinguished with absorption contrast imaging. For example, Figure 4 shows a practical example of a sulfur electrode deposited on a current collector (NWC) and combined with a carbon-binder domain (CBD). From absorption contrast imaging (Figure 4b left), the X-ray absorption of sulfur, carbon, and NWC is very similar, and it is not possible to distinguish these three phases. However, in the phase contrast imaging (Figure 4b right), sulfur shows up brighter and can be segmented easier. Both absorption and phase contrast are combined onto the image shown by Figure 4c. In this image, both sulfur, CBD, as well as current collector can be segmented, and from this image a 3D reconstruction is built as shown by Figure 4a.

#### 2.1.3 Radiation Damage

X-ray imaging is often referred to as “non-destructive”. While this is true in many cases, it is not always the case, and the effects of radiation damage must be considered. This is especially true when imaging at higher resolutions. For example, the dose required to image a sample at a given resolution scales with the fourth power of the voxel size, meaning that a significantly higher dose is required, for example, to image a sample in a nano-CT vs a micro-CT instrument.^41^ The effects of radiation damage are highly dependent on both the sample and a number of experimental and instrumentation factors. Even cell operation history plays a role in the extent of the radiation damage experienced from exposure to the beam.^42^ Studies into the effects of synchrotron radiation on the materials being imaged have been completed to report how X-ray imaging affects materials and cells in operation.^42−44^ These studies are helpful for identifying which materials may suffer more radiation damage than others and possible degradation mechanisms from radiation damage. Of the materials present in electrochemical cells, the ones that are most susceptible to radiation damage are the softer, organic materials like polymers and ionomers.^44^ One important mechanism of degradation that was identified was decomposition of the ionomer in the catalyst layer which led to a decrease in catalyst layer utilization.^42,43^ Under certain operating conditions, such as OCV for a fuel cell, the performance loss from radiation damage can be recoverable; however, this is not the case for every operating condition that may be employed on a beamline.^42^ It is important to be aware of the possible effects of radiation damage because exposure times on the order of hundreds of seconds can lead to adverse effects on cell materials.^43^

### 2.2 Instrumentation

**Figure 5:**
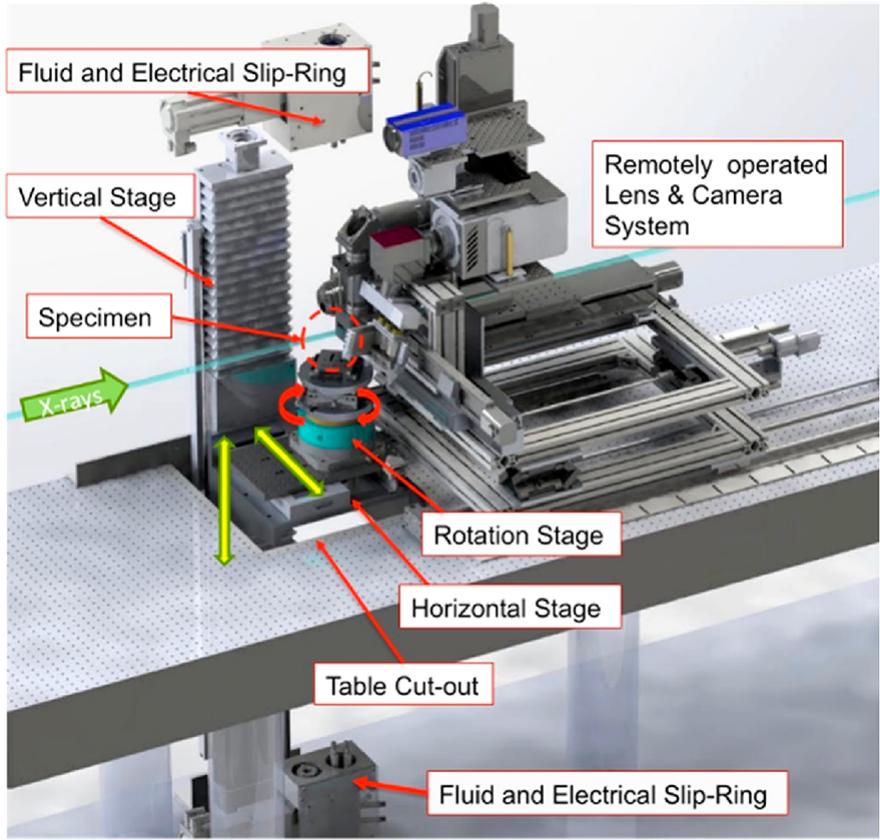
Synchrotron X-ray CT Beamline 8.3.2 endstation at the Advanced Light Source. Adapted with permission from ref (47). Copyright 2016 Society of Photo-Optical Instrumentation Engineers

**Figure 6:**
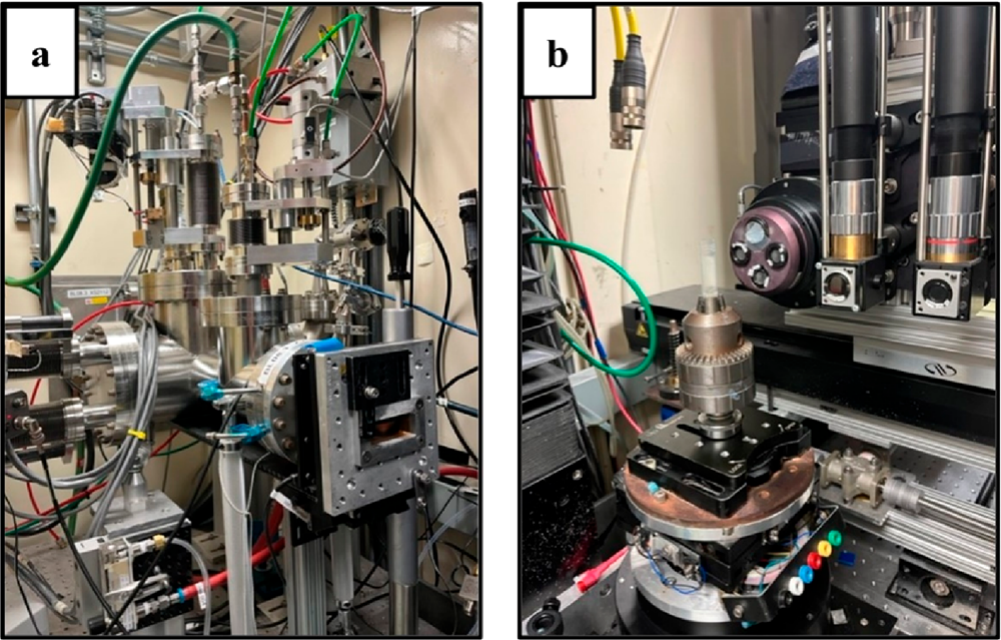
Synchrotron Instrumentation at the Advanced Light Source at Beamline 8.3.2. (a) X-ray photon source. (b) Rotating sample stand and detector system.

Schematic diagram for a micro-CT beamline endstation is shown in Figure 5. The main sections are the X-ray source, X-ray optics, sample control and environment, and detection system. Figure 6a shows photographs of the Kapton window through which X-rays come to the beamline hutch, and Figure 6b shows the rotating beamline stage and detector at ALS 8.3.2 X-ray CT beamline.^45,46^

#### 2.2.1 Source

Synchrotrons produce extremely bright X-ray beams, which enable higher resolution and higher speed scans for nano- and micro-CT experiments than what is available with standard X-ray sources. The X-rays are generated when electrons in the synchrotron storage ring pass through magnetic fields, and the X-ray beams are directed toward “beamlines” with end stations that have the remaining instrumentation needed for CT experiments. Some beamlines have bending magnet sources, which produce X-rays that cover a wide continuous spectrum of wavelengths. Other beamlines have insertion device sources, such as undulators, which consist of a complex array of small magnets that generate a much brighter beam with X-rays concentrated at certain energies, which can be adjusted by adjusting the gap between rows of magnets.

#### 2.2.2 X-ray Optics

In many micro-CT beamlines, the main X-ray optics are part of a monochromator, which allows selection of a particular range of wavelengths. Different monochromators have different “bandpass”, which sets the trade-off between the energy resolution and the flux transmitted through the monochromator. Some beamlines use multilayer mirror monochromators with a relatively large bandpass (the range of wavelengths that pass through for a given setting), while others use Si111 crystal monochromators, which give a smaller bandpass that can be useful for XANES spectral imaging experiments. Many bending magnet beamlines also have a “white beam” mode in which the monochromator is moved out of the way to allow the full spectrum of X-rays to pass through the sample and detector. In some cases, transmission filters or a mirror are used in white beam mode, which can modify the X-ray spectrum. For example, filters can be used to harden the beam by preferentially absorbing longer wavelength (“softer”) X-rays. In some beamlines, additional optics known as compound refractive lenses are used to slightly focus the beam, which yields a larger flux in a given area, allowing for higher speed scans in cases where a larger beam size is not needed.

**Figure 7:**
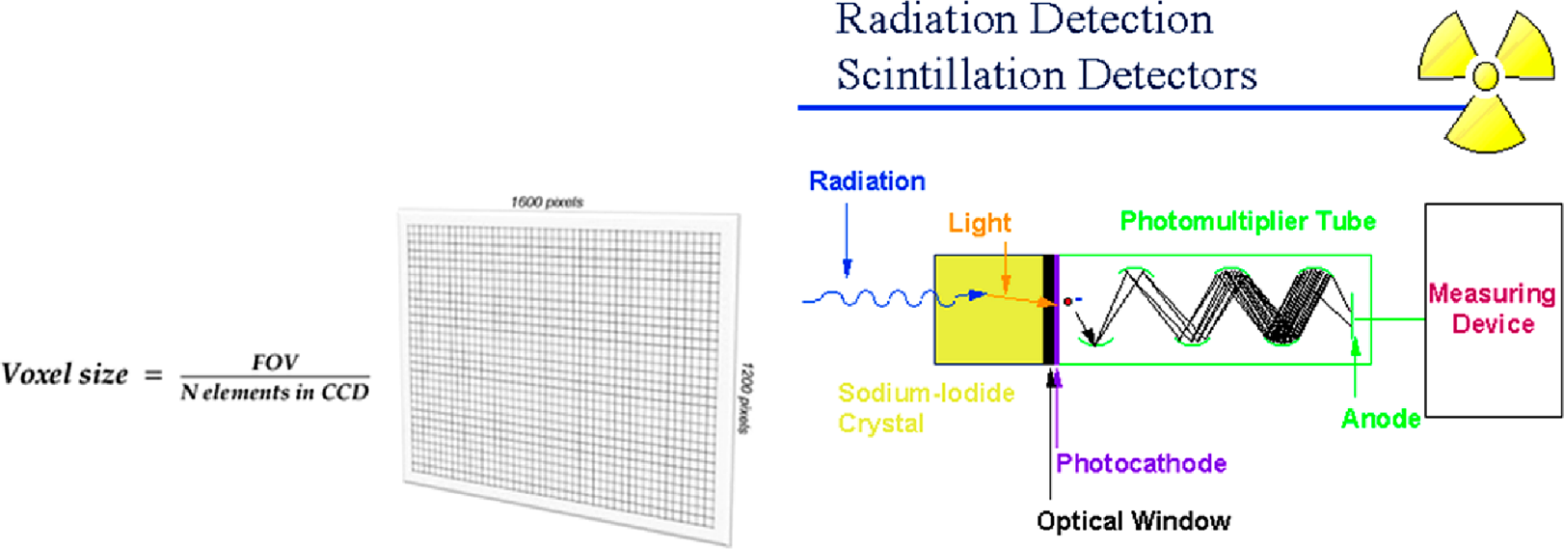
An example of how sodium iodide crystal works as a scintillator.^48^

The resolution at most micro-CT beamlines is limited by the wavelength of visible light (produced by scintillators) to a few hundred nanometers. Figure 7 shows an example of a sodium iodide scintillator and how it transforms X-rays into visible light. When X-rays hit the scintillator, the photons are given off, creating visible light. These photons then strike a thin metal photocathode and continue on to enter the photomultiplier tube (PMT). There, photons hit the photocathode and electrons are ejected. These ejected electrons are then accelerated by an applied voltage to a high energy, and they hit a number of other loose electrons. The signal is multiplied by a factor of a million by the time it reaches the measuring device. For nano-CT, to achieve the higher resolution available at these beamlines, additional X-ray optics are required. These include a condenser optic (either a capillary tube or a zone plate) and an imaging zone plate, and there are often additional focusing mirrors and slits in the beamline to prepare the beam for these optics. In standard transmission X-ray microscopes, the resolution when using a zone plate lens is limited by the width of the outermost zone. In most cases, this is in the range of tens of nanometers. Because hard X-rays require zone plates that are microns in thickness, these zone plates have extremely high aspect ratios and are challenging to fabricate.

Some beamlines have developed a scanning microscopy method called ptychography which uses a lower-resolution zone plate combined with collecting diffraction patterns at a series of positions and performing data processing in a way that they are able to collect images with resolutions better than the limit of the zone plate resolution.^49−51^

An alternative approach to achieve nanoscale resolution is to focus the beam to a small spot with, for example, Kirkpatrick–Baez (K–B) mirrors, place the sample after the focus spot (in a region where the beam is expanding in size), and place the detector at a distance back which allows the image to be magnified (this gives phase images).^52,53^ For phase imaging in nano-CT, phase ring optics are utilized in fuel-cell applications for materials with especially low *Z*-numbers.^54−58^ For micro-CT, gratings and other optics are sometimes used to accomplish phase imaging.^58,59^

#### 2.2.3 Sample Control and Environments

The sample stage stack can generally be moved to position the sample (or a portion of the sample) in the beam and can then rotate the sample during a scan. Because of the high flux available at synchrotrons and the high speed of detectors available, most beamlines collect scans in a mode known as a continuous, fly, or flying scan. In this mode, the sample is continuously rotated at a constant speed, while images are collected.

To achieve resolution in a 3D reconstruction that matches the resolution available in a 2D image, stability is critical. In some cases, stages are available that position the sample accurately throughout the scan to within the resolution element. However, in some nanoimaging instruments, the sample position is not controlled to within the resolution, and in these cases an additional image processing step is required in which the images are aligned to a common frame of reference and the undesired shifts and rotations are removed.

Doing in situ experiments that require sample environments leads to challenges for the sample positioning system. Sample environments often lead to increased weight, which increases the challenge of finding sample positioning stages that can handle both the positioning accuracy and weight. In addition, sample environments (described in a later section) often require electrical, liquid, and gas flow, and the supply lines for these components can interfere with the rotation of the sample and in many cases can lead to the lack of accurate positioning of the sample throughout a scan. To allow the use of sample environments and minimize the problems of supply line interference, in many cases, electrical and hydraulic slip-rings are used below or above the sample.

#### 2.2.4 Detection

Detection is most commonly performed with a scintillator and a visible light detector. The scintillator absorbs X-rays and emits visible light, which is imaged with a visible light microscope and detector, as shown by Figure 6. Many beamlines currently use CMOS detectors because they allow high-speed readout. These detectors generally have pixel sizes of 6 μm or smaller, and so optical microscope objectives with magnifications between 1× and 40× yield effective pixel sizes between ∼5 μm and ∼100 nm. Higher-magnification lenses generally have a smaller depth of focus, which sets the limit of the thickness of the scintillator. Scintillators with thicknesses between 5 and 200 μm are used, and common scintillator materials include YAG (yttrium aluminum garnet), LuAG (lutetium aluminum garnet), and GGG (gadolinium gallium garnet) crystals which are doped, commonly with cerium. Scintillators are chosen to have a very high light conversion efficiency and low ghosting. In addition, they must be free of defects, scratches, and contamination because imperfections on the scintillator are a major source of artifacts in images. Microscope objective lenses are commonly used for magnification (such as Olympus or Mitutoyo lenses), but some beamlines also use custom lenses with very high numerical apertures which allow maximum light throughput and thus maximum scan speed.^16^

## 3 General Aspects for Technique and Image Processing

### 3.1 Reconstruction

What is desired in computed tomography is to perform “tomographic reconstruction” to derive from the raw data the 3D distribution of attenuation coefficients in the sample.

#### 3.1.1 Normalization

The “raw data” in a tomography scan consist of a series of projection images (often called radiographs) collected as the sample is rotated to different angles. In the simplest case of pure attenuation contrast, as described above, these images are composed by a series of pixels which represent the line integrals of the attenuation from the materials through which the beam passes. The measure of the degree of X-ray penetration into the material is described by the X-ray attenuation coefficient. Based on the Beer–Lambert law (see section 2.1), the X-ray attenuation coefficient, together with the distance of the X-ray pathway inside the material (material thickness), decides the transmitted portion of the beam (i.e., transmission) for a specific beam angle. In practice, the X-ray illumination is not perfectly uniform, so in addition to collecting these projection images measuring attenuation contrast, additional images are collected with the sample moved out of the way; these are known as flat, bright, or white fields, or background images. These allow correction for inhomogeneous X-ray illumination. A final set of images is collected with the X-ray shutters closed, often known as dark fields; this allows correction for the dark current in the detector. Multiple bright and dark field images are collected to allow them to be averaged (or combined by calculating the median) and, thus, reduce noise. The sample (*S*), average bright (*B*), and average dark (*D*) field images are combined to yield a percent transmission image (*T*):
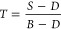
3

#### 3.1.2 Phase Retrieval

For pure attenuation contrast CT, these percent transmission images are used for subsequent steps. However, in many cases an image has contributions of both phase and attenuation.^60^ In these cases, it may be desirable to isolate either the phase or the attenuation component of the complex index of refraction. In these cases, the “phase retrieval” process is applied to the image to separately yield the phase component (or the attenuation component) from the original measured percent transmission. The phase retrieval approach depends on the data collection approach and on which assumptions can be applied to the situation (for example, how many materials are present).^61−63^ Similarly, applications utilizing phase retrieval have been utilized with lab-based and synchrotron sources.^64,65^

#### 3.1.3 Tomographic Reconstruction

**Figure 8:**
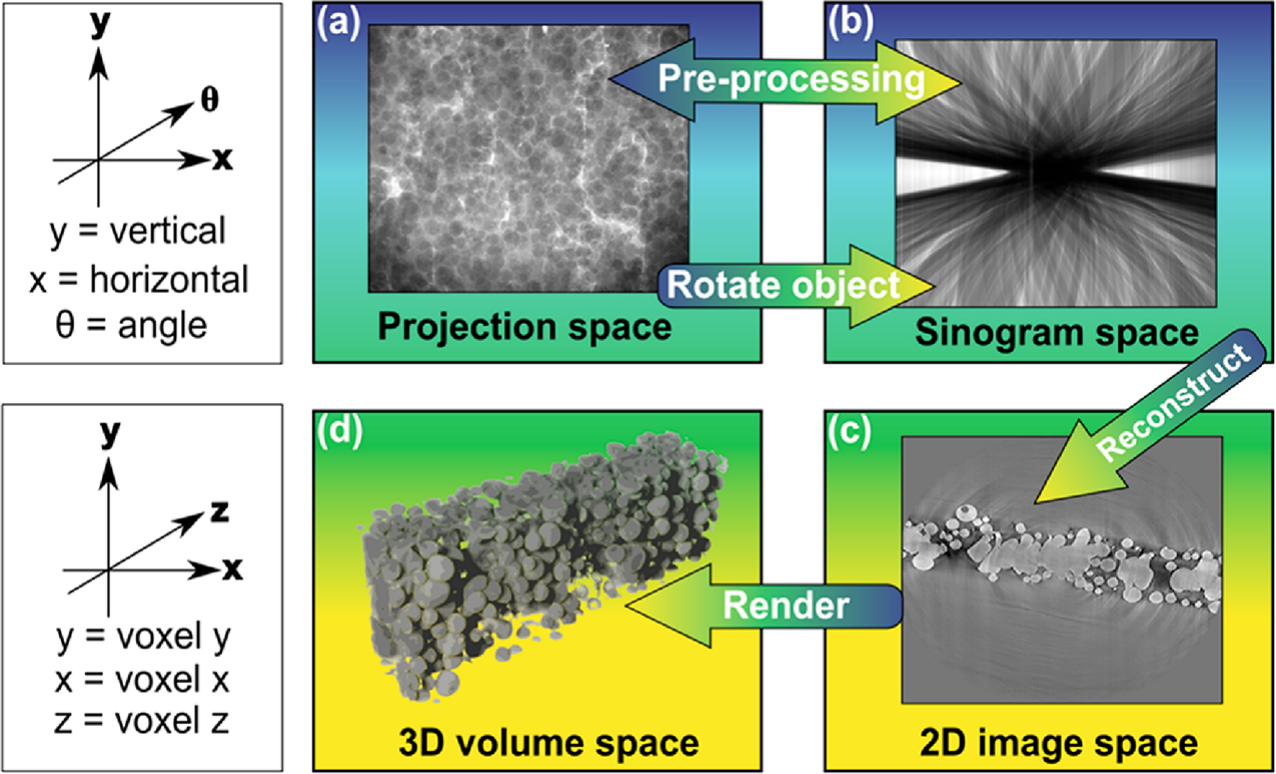
Workflow and visualization of tomography data processed through each data space. The axes on the left correspond to the rows of images they are next to. Moving from the projection space (a) to the sinogram space (b) involves taking a single row of pixels over the entire series of angles from (a). Going from (b) to the 2D image space (c) requires reconstruction using analytical or iterative methods. Going from (c) to the 3D volume space (d) involves applying image analysis techniques onto the entire 2D image stack produced from the reconstruction techniques.

The determination of the 3D distribution of attenuation coefficients is most often done on a slice-by-slice basis. A “sinogram” is the term given to the image formed by taking a single row of pixels from the projection images over the series of angles used during a tomography scan, as shown in Figure 8. The Radon transform is the mathematical description of the relationship between a sinogram and a digital slice through the sample. In practice, there are a number of approaches which are used to generate digital slices from sinograms, and these are often divided into analytical methods (such as filtered back projection and the GridRec method^66^) and iterative methods such as ART, SIRT,^67^ as well as model based iterative methods (MBIR).^68,69^ Iterative methods are more computationally intensive but can generally yield superior results. The difference is most apparent in cases where there is undersampling, for example, when a small number of projection images are used. This might be done to speed up the scan time for an in situ experiment. There are also approaches specifically developed for phase contrast CT imaging.^70,71^

Several software packages are available that implement different algorithms. TomoPy is one popular package that makes various algorithms available through straightforward python wrappers. ASTRA Toolbox^72^ is another popular package, including through the TomoPy wrapper.^73^ SVMBIR^74^ is a package for MBIR.

In the past few years, machine learning approaches have also started to gain more widespread adoption. Some of these approaches have focused on, for example, denoising or upsampling either projection images or the final reconstructed images.^75^ Others have focused on enabling higher-speed scans with fewer projections by training models based on static scans of a sample prior to beginning an in situ experiment, and then using those models during image reconstruction and recovery of the high-speed scans.^76^

#### 3.1.4 Center of Rotation and Image Alignment

One of the inputs for tomographic reconstruction algorithms is the location within the image of the axis of rotation. The axis of rotation is not necessarily consistent between scans, whether due to instabilities in the equipment or changes in the setup (sample position, resolution, etc.) and so is often determined after the scan during the tomographic reconstruction process. There are a number of methods to do this automatically (for example, multiple methods in TomoPy), though in some cases these methods fail, most often due to artifacts in the images or to low sample image contrast, in which case the center of rotation is sometimes found by displaying a difference map of the projection image at 0° with a flipped version of the projection image at 180°; when the 180° image is flipped around the correct axis of rotation, the difference between the two images should be 0. Alternatively, a series of reconstructions can be carried out using different test values for the center of rotation, and the best one can be determined by a visual inspection of the reconstructed slices. Incorrect center of rotation leads to characteristic crescent patterns and streaks in reconstructions. For nano-CT, in some cases, a more complete alignment needs to be carried out to correct for jitter or drift in the sample position during a scan.

#### 3.1.5 Artifact Reduction

**Figure 9:**
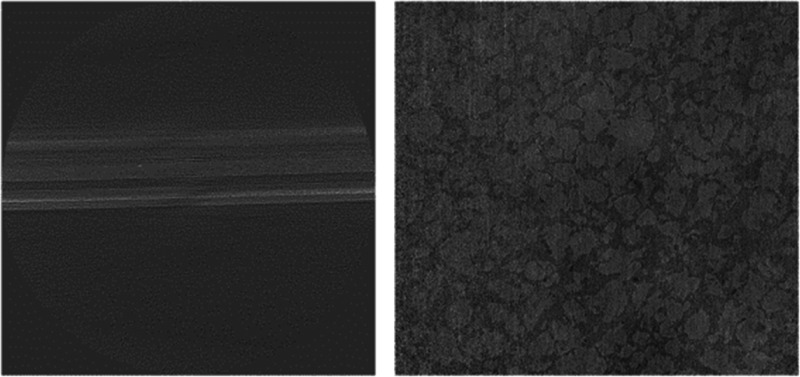
Mottled/low contrast scan, low spatial/high temporal resolution scan.

A major consideration during tomographic reconstruction is artifact reduction. Some sources of artifacts are zingers (when X-rays directly hit the CMOS detector without being converted by the scintillator), distortion in the lens, shifting of the X-ray illumination, and imperfections in the scintillator, leading to ring artifacts. There are various approaches to reducing each of these, some of which are in TomoPy and some of which are available in various other packages, for example, Algotom.^77^ In addition to artifact reduction, noise reduction can often be important. For time-resolved experiments, each scan must be short enough to achieve the desired time resolution, and in many cases, this means using exposure times or a number of angles smaller than what would be used for optimal imaging of a static sample. As shown in Figure 9, this can lead to low-contrast, higher-noise scans.

### 3.2 Image Processing

Image processing is an essential step in extracting useful information from CT data. Here, we introduce the operations done on the imaging data set after reconstruction. Many of the image processing steps, like ring removal and denoising, have been incorporated into the reconstruction algorithm already and do not need to be done separately.^78^ This benefits the CT users and shortens the analysis time after collecting and reconstructing the CT data. However, the processing of the reconstructed data is still necessary, and researchers need to decide what processing techniques to use based on their data and needs, as there is no universal recipe because image processing is highly dependent on data under study.

The typical image processing process includes the following steps: desired view and ROI locating, contrast stretching, denoising, and image segmentation (discussed in the next section). If the data set is from in situ experiments, image registration (alignment) is essential. The procedure mentioned above is for reference, and it is expected to be changed based on the data and needs.

**Figure 10:**
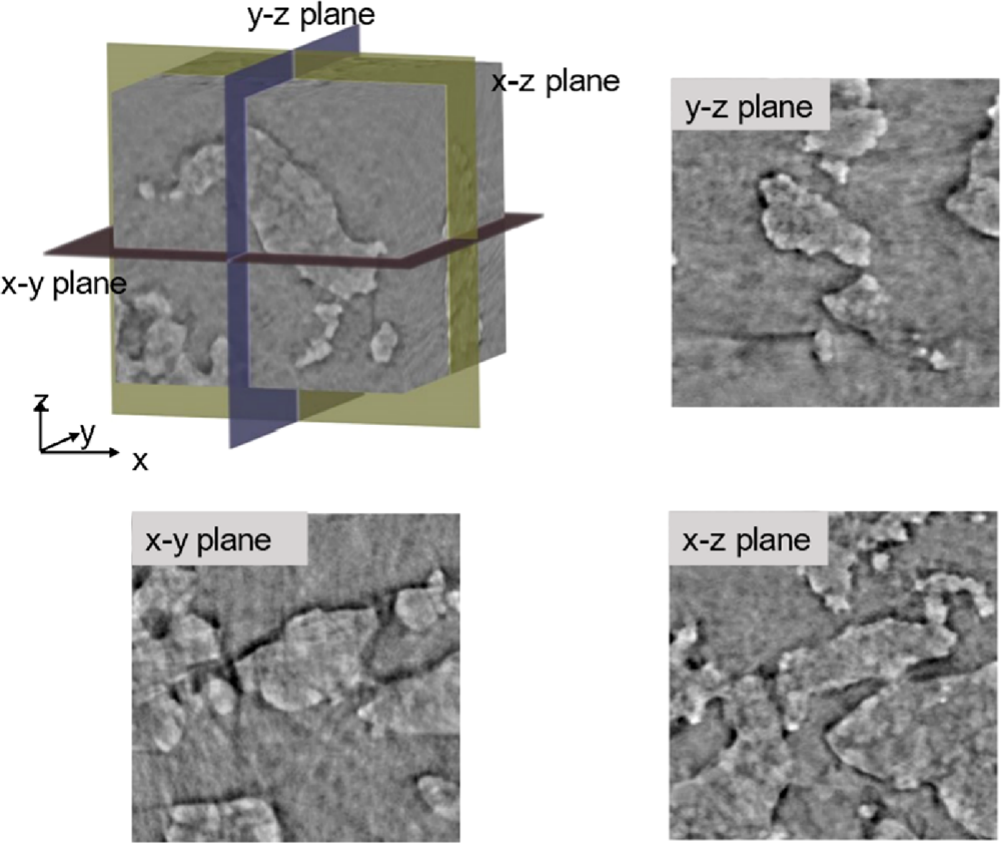
XYZ coordinate system and images are represented in the *x*–*y*, *y*–*z*, and *x*–*z* planes.

CT data contains three-dimensional information. A stack refers to the whole data set (a set of images), while the slice is the image shown as the main view. The data stack is demonstrated in the XYZ-coordinate system. That is to say, the view of the data slice can be shown in two-dimensions in XY, YZ, and XZ planes which is shown in Figure 10.

Reslice is a transformation for 3D imaging data sets that rearranges the data and shows another plane as the main 2D view. By reslicing the CT data stack, the data set is rotated, and thus, the main view is changed. This helps researchers find the desired view. Another benefit of this transformation is that it helps researchers locate the ROI from 3D views. When cropping the data set to make the ROI, researchers need to crop and check on at least two different views to make sure the selected ROI is the one that was intended. During this process, some image transformation operations such as rotation should be performed when needed.

The goal of manipulating contrast stretching is to acquire image data with better contrast. By applying the appropriate contrast stretching function, the pixels with an intensity lower than a threshold value would be darkened, while the others would be brightened. This allows one to adjust the grayscale values of the images.

**Figure 11:**
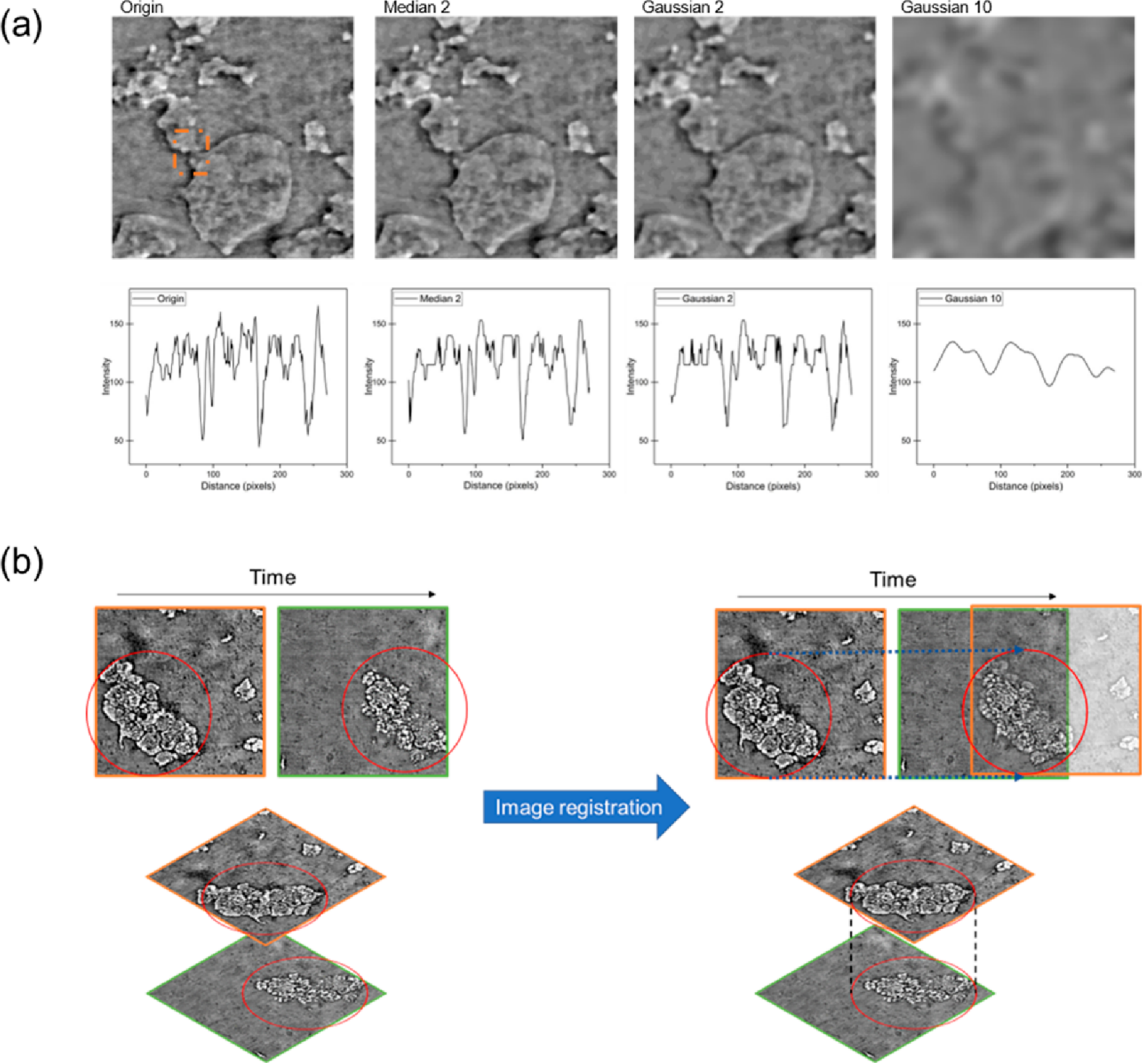
Examples of image filters and image registration. (a) Filters commonly used in image processing such as Median and Gaussian. The number after the filter name is the radius of smoothing. The bottom row shows the line profile of the chosen area (marked by an orange rectangle). (b) An example of a scheme of image registration.

The denoise command is used to remove the salt and pepper noise of the image. It usually is achieved by applying filters, including linear filters, such as mean filters, and nonlinear filters, such as median filters. This operation is performed with some extent of edge smoothing. The resulting image is less sharp than the original one, but it eliminates the salt and pepper noise. Therefore, the denoise filter must be carefully chosen. Figure 11a shows examples of the resulting image from two common filters provided, both with radius of smoothing of 2. It is also worth noticing that there are sharpening filters to highlight the transitions of the intensity inside the images.

Image registration (alignment) is a way to align multiple images based on the same or similar spatial features. For the data coming from in situ experiments, by comparing the same feature, it is expected to observe the morphological transformation that happened during the in situ experiment. The registration can be done by simply translating one image, operating both translation and rotation, and combining the above two while scaling and shearing. Some intensity-based algorithms, like the bilinear interpolator, are also available. Since in situ experiments are done with the same setup and without manually moving the samples, a simple algorithm that can perform translation and rotation should be sufficient for handling the in situ CT data. Figure 11b demonstrates one scenario in which image registration was used. An example is the data obtained in an operando experiment, showing the dendrite evolution during battery discharging. The sample was not moved by researchers during the experiment, but the data set obtained in two different timesteps was not aligned because of expansion of material during charging/discharging. For extracting the same ROI and for better observation of the evolution of the dendrites, image registration was conducted, and the images were then put in the same coordination system as shown by Figure 11b.

A commonly used tool for analyzing CT data is ImageJ. Fiji distribution of ImageJ is an open-source software with plenty of plugins for image analysis.^79^ Commercial CT analysis tools, including Dragonfly (Object Research Systems), AVIZO (Thermo Fisher Scientific), VG Studio (Volume Graphics), SimpleWare (Volume Graphics), etc., are also available for more convenient comprehensive analysis.

### 3.3 Segmentation

Segmentation is essential to analyzing CT data, enabling quantitative analysis. Semantic segmentation is the typical segmentation done on CT data, which labels every pixel in a cross section to a class. In this way, each phase of the samples can be extracted and separated out from the whole data set. Multiclass semantic segmentation is sometimes needed, as there might be multiple phases present within the samples.

Thresholding is the simplest way to achieve segmentation. It sets a thresholding number of the pixel values, putting all of the pixels with the value below the number into one class and the other pixels into another class. If more than two classes exist in the data, then more than one thresholding number is needed. This method might fail when the contrast between the two phases is poor and the two phases are overlapping in contrast. The simple thresholding cannot satisfy the needs of the more complex data sets. Some other traditional ways to do segmentation include watershed,^80−82^ K-mean (clustering-based),^83−85^ and canny (edge-detection).^86−88^

**Figure 12:**
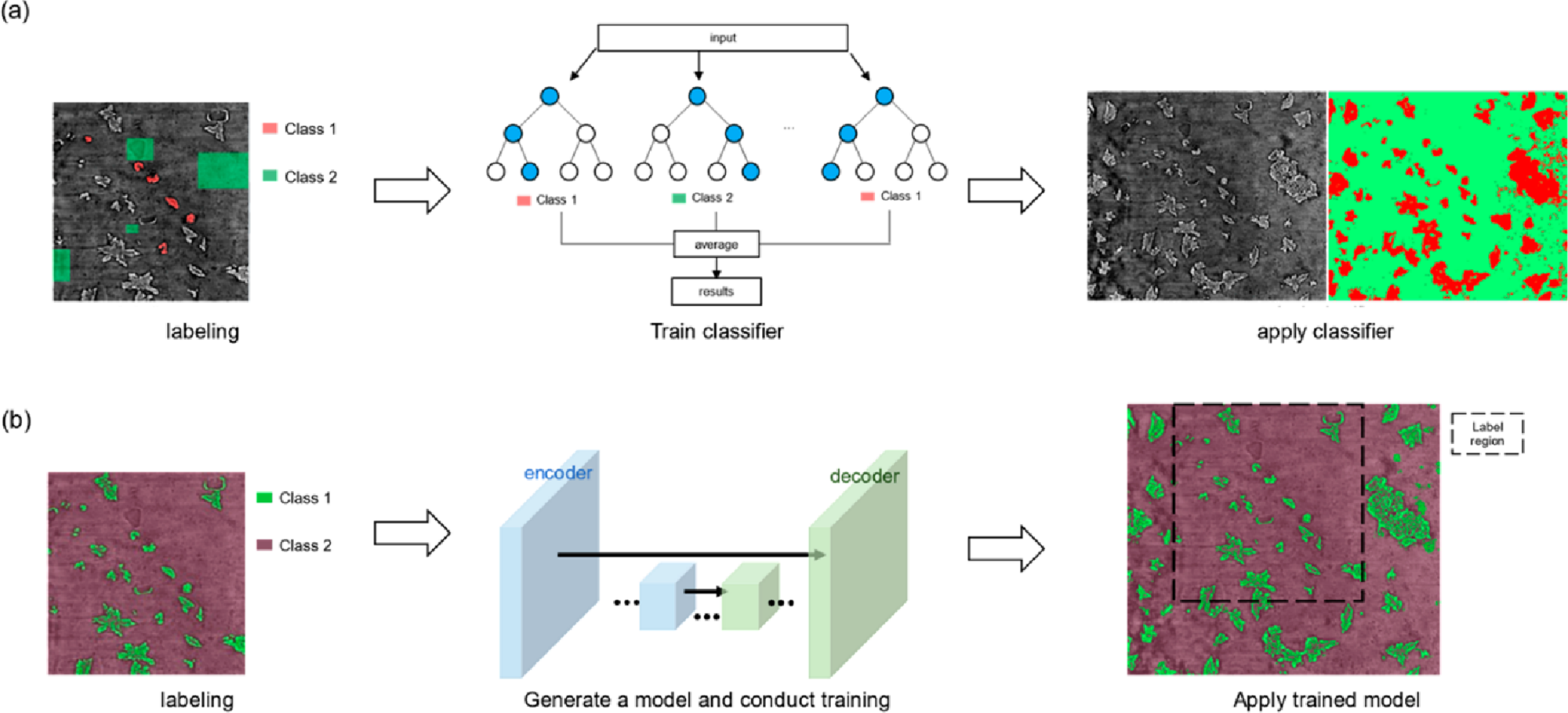
Scheme showing the workflow of segmenting image in (a) machine learning (using random forest model as an example) and (b) deep learning (using U-Net as an example).

Machine learning is intensively used to produce high-quality segmentation X-ray CT data, which includes utilizing machine learning classifiers.^89,90^ With the progress in the computing power, more studies took advantage of the convolutional neural networks when conducting image segmentation for X-ray CT data.^91−94^ The methods that are built based on neural networks are referred to as deep learning. Deep learning, though a subtype of machine learning, is commonly discussed separately from machine learning. Deep learning methods are always used to deal with larger and more complicated data sets. However, they require a high-performance GPU and expect larger amounts of labeled data as input. Typical deep learning models used in CT image segmentation are U-Net^91^ and its modified models.^92,95,96^ There are many toolboxes allowing researchers to conduct segmentation with machine learning. In Figure 12, the simplified workflows of the segmentation with machine learning (using a random forest model as an example) and deep learning (using U-Net as an example) are shown.

Building a metric to evaluate the segmentation results can also be important. This helps researchers to understand the uncertainty of the quantitative analysis done on the segmentation.^97−99^ Usually, there are four possible outcomes regarding the segmentation result of a specific class: (1) true positive, which is the pixel number of the correctly labeled pixels; (2) false positive, which is the pixel number of wrongly labeled pixels; (3) true negative, which is the pixel number of the correctly unlabeled pixels (labeled as another class); and (4) false negative, which is the pixel number of the wrongly unlabeled pixels. Most of the evaluation metrics are based on the above outcome. Recall is used to represent how successful the results are in identifying one specific class. Precision represents how pure the segmentation result is regarding one specific class. Accuracy is the correct rate of the segmentation. Intersection over union (IOU) evaluates the overlap of the ground truth (labels) and the segmented region. The equations for the above-mentioned metric are as follows:
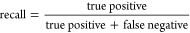
4
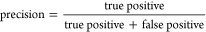
5
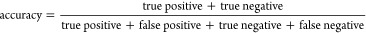
6
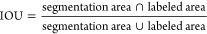
7

### 3.4 Quantitative Information

The reconstructed image post processing can involve extraction of various quantifiable data of interest. For electrochemical systems involving porous media, for example, morphological properties such as porosity, tortuosity, pore size distribution, etc., are often quantities of relevance. The first step toward obtaining this quantitative information is to segment the image by applying proper threshold values manually or by using machine learning algorithms as discussed in the previous section. The subsequent accuracy of the results depends on how well the threshold is set. ImageJ is the most popular image processing software capable of extracting multitudes of information for practically all applications and data sets with a growing library of plugins that users from all over the world write and publish as open-source scripts. Other software packages for image processing include Dragonfly, which is popular for research, and VG Studio Max, which is popular for industrial applications.

For porosity measurement in three-dimensional volume, the measurement theory is illustrated in detail by Taud et al.^100^ The porosity can be simply defined by as
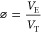
8where *V*_E_ is the volume of empty space and *V*_T_ is the total volume of the porous media. In image processing, porosity is often calculated as a function of distance from the point of measurement by calculating the area fraction of the void phase over total area per slice in the image stack.^7,12,101−105^ Several studies have reported calculation of pore size distribution by micro X-ray CT image processing techniques.^106^ The pore size can be defined as the diameter of the largest sphere that fits inside a 3D pore volume and contains the point *p⃗* such that
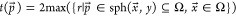
9where *t*(*p⃗*) is the local thickness inside an object Ω. sph(*x⃗*, *y*) is the set of points inside a sphere with center *x⃗* and radius *y*.^107,108^ This method can also be applied for measuring particle size distribution in an image data set obtained from various imaging techniques, for example, quantifying dendrite size and form factor in Li-ion batteries or ellipsoid factor of compressed GDLs in fuel cells.^109−111^ Another important quantity of interest related to porous media in electrochemical devices is tortuosity. It quantifies the degree of diffusive transport resulting from convolutions of flow pathways in porous media. This information can be used to quantify mass transport resistances and relate them to system polarization curves. Tortuosity factor, τ, can be simply defined as the ratio of the actual path length (*L*_a_) to the Euclidean distance (*L*).
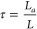
10Alternatively, tortuosity is also defined as
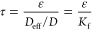
11where ε is the porosity, *D* is the intrinsic diffusion coefficient, *D*_eff_ is the effective diffusion coefficient, and *K*_f_ is the formation factor. For tomography data processing, these values can be obtained with Lattice Boltzmann Method and CFD, but the simulations are often tedious and time-consuming.^112^ The TauFactor application in Matlab is an efficient and fast calculator for tortuosity factor from tomography data, and it is a popular choice among the community. TauFactor can analyze image volumes larger than 10^8^ voxels on a single microprocessor core in a few hours.^113^ These techniques have been used in alternative ways for quantifying membrane and catalyst degradation or membrane electrode assembly (MEA) cracks in fuel cells.^114−117^ Some of the other quantitative information that can be obtained includes surface area measurements of MEAs,^118,119^ water phase quantification in GDLs of fuel cells,^111,120−122^ quantification of oxygen flow in PTLs of PEM water electrolyzers,^123,124^ and estimation of electrochemically active area for PEM electrolyzer anodes by quantifying the triple phase contact area (TPCA) formulated by Kulkarni et al.^101^
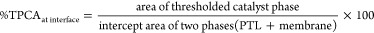
12The respective areas of the catalyst and the other two phases are obtained by thresholding separately and then measuring the area within the defined interface.

## 4 Applications of X-ray CT to In Situ and Operando Studies in Electrocatalysis

Several works^14,125^ provided an extensive overview on nano X-ray CT use for battery materials. Compared to battery studies, very few in situ and operando X-ray CT studies have been carried out for electrocatalysis applications. This is mainly because many of the questions that the field of electrocatalysis tries to answer relate mostly to the surface of the nanoparticles or other catalysts. For batteries, many of the particles are micron scale and the questions that one tries to answer relate to bulk phenomena (particle cracking, particle morphology, binder distribution, etc.). Being resolution-limited (∼30 nm), nano X-ray CT had limited applicability to the field of electrocatalysis. Recently, as one integrates electrocatalysts into catalyst layers and electrodes, the morphology of electrodes and uniformity of the distribution of electrocatalysts, as well as understanding the distribution of the other phases in the electrodes, gained more importance. Thus, nano-CT and in many instances even micro-CT became essential tools to answer the questions related to the morphology of the catalyst layers. In addition, many beamlines currently combine X-ray CT and other techniques, such as X-ray absorption spectroscopy (XAS), XRF spectroscopy, XRD, and some other techniques that allow multimodal interrogation of combined morphology, structure, and chemistry.^126,127^

### 4.1 Overview of Nano-CT Beamlines

Mostly nano-CT beamlines are used for electrocatalysis studies, as they have nanoscale resolution compared to the microscale resolution of micro-CT. Here, we will overview several relevant nano-CT beamlines and their capabilities for multimodal imaging and in situ/operando environments. European Synchrotron Research Facility (ESRF) beamlines ID16A^128^ and ID16B^129^ are nano-CT beamlines. ID16A Nano-Imaging beamline combines nano-CT and XRF.^126^ It allows for phase-contrast imaging, enabling imaging of soft materials, and it operates at higher energies of 17–33.6 keV with resolution of 30 nm.^126^ ID16B Nano-Analysis beamline combines nano-CT with XRF, XRD, and XAS techniques and allows for in situ and operando sample environments.^127^ The energy range of 6–65 keV and beam size of 50 nm allows for various imaging modes.^127^ However, the X-ray CT mode is limited to several selected energies. The main advantages for electrochemical technologies and electrocatalysis of these beamlines are that they operate at higher energies, lowering damage to soft materials. At higher energies, less X-ray absorption occurs, meaning less beam damage is done to soft materials.

NSLS II 18-ID^130^ full field X-ray imaging (FXI) features an energy range of 5–11 keV and a very fast imaging time of about 1 min per scan, achieving a spatial resolution of 30 nm.^131^ This beamline also allows for 2D and 3D XANES, in addition to transmission X-ray microscopy (TXM).^131^ The beamline currently does not have the capability for phase-contrast imaging. In situ cells that are stationed at this beamline include electrochemical testing and hot cells.

APS 32-ID^132^ full field imaging and transmission X-ray microscopy supports two experimental stations: 32-ID-B and 32-ID-C. 32-ID-C is designed to be a high-speed microscopy beamline with 2D imaging.^133,134^ 32-ID-B features phase-contrast at 8 keV energy, 30 nm resolution, and has also XANES technique capabilities.^134^ The imaging time is a bit slower than that of the NSLS II 18-ID. The beamline also has in situ electrochemical and hot cell capabilities.

SSRL 6-2c^135^ beamline supports nano-CT and XAS. The resolution is 30 nm, and a variable energy range can be selected from 2.36 to 17.5 keV.^136^ The beamline features only the absorption mode and has various in situ environments, including electrochemical cells, gas cells, and hot cells.

SPring-8 Beamline 20XU^137^ operates in energy range of 15–37.7 keV and has a resolution of 75–100 nm.^138^ This high-energy beamline combines both micro- and nano-CT capabilities and has a scan-time for nano-CT of 30–60 min.^138^

### 4.2 In Situ Studies

In situ studies are performed to collect data for catalyst behavior in conditions that are similar to actual operation conditions.^28^ These studies are helpful to understand how catalysts and other materials will behave in the cell for which they are designed and how their structure is affected by use. Some techniques for these kinds of tests involve preparation steps ex situ before measurements are taken in situ. In situ tests are helpful for catalyst synthesis, because porosity, pore size distribution, and tortuosity are very important parameters for catalysts that can be measured with X-ray CT. X-ray CT can also be combined with other imaging techniques, so that more focused data can be gathered.

#### 4.2.1 From Ex Situ to In Situ

**Figure 13:**
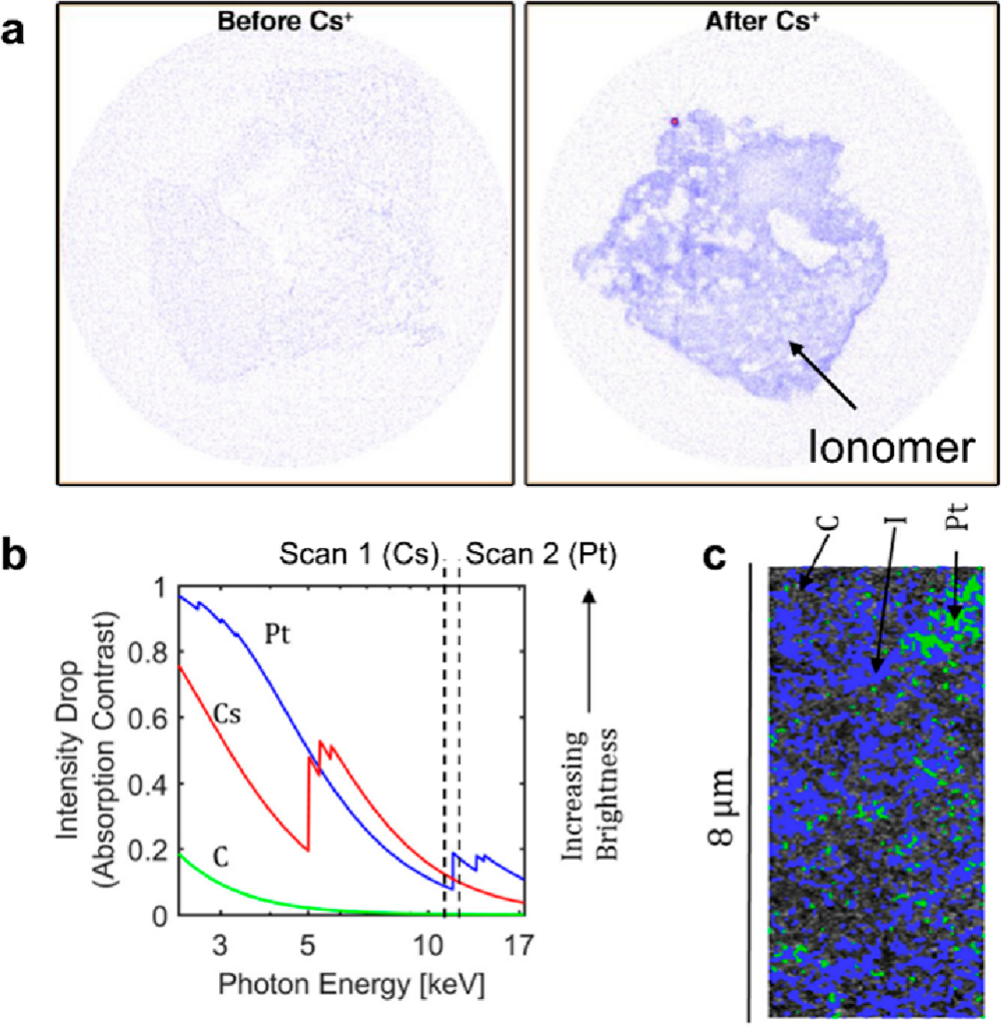
Examples of Cs-ion staining of ionomer for ionomer visualization of the catalyst layer within fuel cells. (a) Contrast difference for PGM-free catalyst layers with and without Cs-ion exchanging of ionomer. Adapted with permission from ref (139). Copyright 2016 American Chemical Society. (b) Optical properties of Pt, Cs, and C over the energy range available at 6-2 beamline at SSRL. Normalized X-ray intensity decreases of Pt, Cs, and C in absorption contrast. (c) Cross section tomography image of Pt, ionomer, and carbon. Adapted with permission from ref (141). Copyright 2019 Elsevier.

Several studies mapped ionomer distribution in the catalyst layer using heavy staining elements, such as cesium.^139−141^ This method works well for materials or catalyst layers where there are no other heavy elements present, as Cs will be the most absorbing element in the structure. However, many of the electrocatalysts are heavy elements, such as Pt or Ir, and the X-ray absorption from Cs and from these elements can give similar contrast. As shown in Figure 13, Normile et al.^141^ used pre- and post-Pt edge to map ionomer stained with Cs and Pt distributions within the catalyst layer. This simple two-energy imaging allows us to differentiate the two elements. It can be done only at the beamlines, as currently, commercial nano-CT scanners do not have more than one anode and thus operate only at one monochromatic energy.

For electrolyzer catalysts, mostly ex situ work has been done using tomography. Leonard et al.^102^ has shown 3D distribution of IrOx within the catalyst layer, showing significant agglomeration of large particles. Zaccarine et al.^142^ used various characterization techniques including 2D TXM mosaic maps to show Ir and IrO_2_ distribution within the proton exchange membrane water electrolyzer catalyst layer, combined with XANES maps available at SSRL 6-2 beamline.

#### 4.2.2 X-ray CT Used for Catalyst Synthesis Studies

Electrocatalysts are at the heart of many electrochemical devices including fuel cells, electrolyzers, redox flow batteries, and others. Under acidic conditions, when proton exchange membrane (PEM) is used, many of the transition metal catalysts are unstable, and PEM-based devices rely on precious group metal (PGM) catalysts, such as platinum, iridium/iridium oxide, and ruthenium. The design space for these electrocatalysts includes an increase of surface area because the reactions are heterogeneous; they take place at the catalyst–electrolyte interface. Another factor to investigate is the catalyst activity. An increase in their activity can occur through shape-control or alloying.^143,144^ Another strategy is to use atomically dispersed catalysts, such as Fe–N–C catalysts for ORR that showed significant promise for catalyzing various reactions.^145,146^ The process of synthesis for Fe–N–C is challenging, including mixing of iron salts, nitrogen-rich precursors, and solvents to heat treat the mixture at high temperatures to form a carbon network and to disperse iron atomically to form catalyst sites. Studies determine the temperature profile andfinal synthesis temperature empirically, while correlations between process synthesis parameters and the final activity of catalysts is not well-understood.^147,148^ X-ray CT can be used to address some of the questions related to the catalyst synthesis process, including morphological changes, material transformations, and other phenomena.

**Figure 14:**
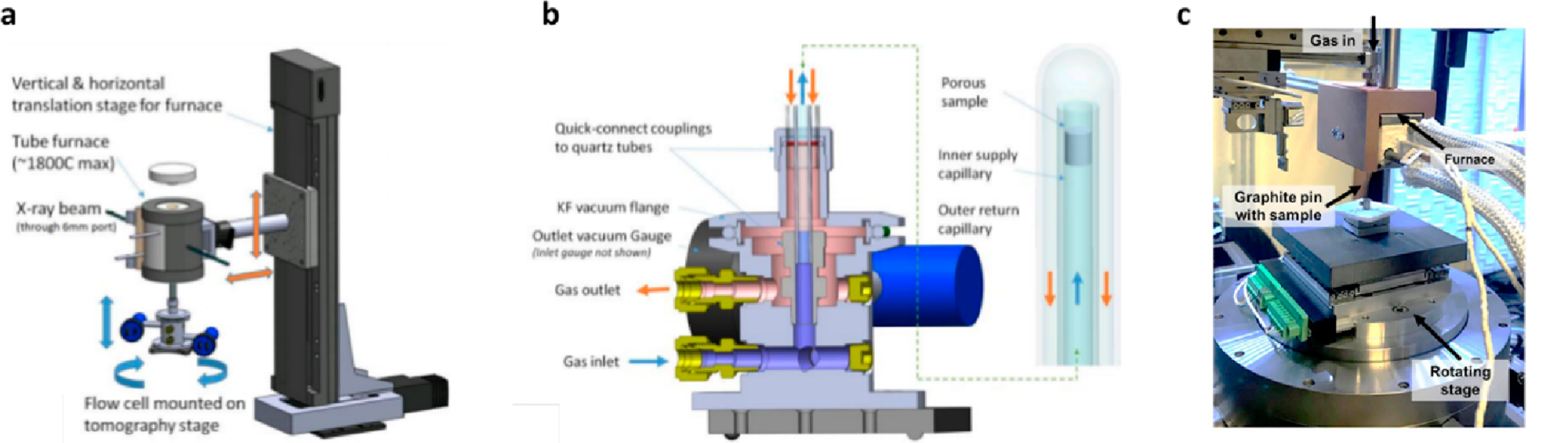
In situ furnace system for CT. (a) The structure of the high-temperature environmental cell used in micro-CT experiment. (b) Scheme of the system for high-temperature micro-CT. (c) Furnace setup for high-temperature nano-CT experiment. Panels a and b adapted with permission from ref (153). Copyright 2018 Cambridge University Press. Panel c adapted with permission from ref (154). Copyright 2021 Elsevier.

Pyrolysis is the most common method to synthesize catalysts. Through high temperatures, atomic bonds rearrange, new ligands form, some materials evaporate, and the elements might redistribute. All these changes would be reflected in the morphology of the final sample.^149−152^ For these pyrolysis experiments, the furnace must be able to control the temperature accurately. Also, the design of the furnace must be fitted for both the heating experiments and the CT experiments. It must include an X-ray transparent window to allow for data to be collected. The samples must be inside the atmosphere for most of the pyrolysis experiments. Thus, the gas must be able to flow in and out. For micro-CT experiments of pyrolysis of catalysts, the samples were mounted on the top of a ceramic holder which was placed inside a quartz tube as shown in Figure 14a.^153^ The gas can be purged inside this quartz tube, as shown in the schematic of Figure 14b.^153^ The quartz tube was mounted in an airtight seal onto a base. The holder and tube were placed on a rotating stage. The top part of the holder and the tube were enveloped by a furnace, so that the samples can be heated.^153^ It is worth noting that there could not be any intense movement of the samples during the scanning. The movement might cause the rotating center to be unfindable, which renders the data reconstruction difficult or unable to be completed. This is a very strict requirement for nano-CT, since a light movement at the bottom can translate to an intense movement at the top where the sample is mounted. For the in situ nano-CT experiments, the materials were mounted on the top of a graphite pin as shown in Figure 14c.^154^ The reason for using the graphite pin is because the graphite has a low thermal expansion coefficient at high temperatures, so the movement of the samples can be reduced. The pin was mounted on a high-accuracy air-bearing rotary stage. The furnace enveloped the top part of the pin, and the gas was flowed in and out through the furnace. To avoid thermal fluctuations, a preheat tubing segment was used to heat the gas entering the furnace;^154^ thus, the movement caused by heating was further limited during the pyrolysis.

Morphological properties, including surface area, porosity, and tortuosity, are critical to catalysts in an electrochemical device.^155−157^ These properties are related to the diffusion and dissolution of the reactants and products inside the catalysts’ structures. The morphological properties of operating devices can be linked to the electrochemical performance, and many studies have done so.^29,158−161^ Though useful, more attention is needed to improve the catalyst design and synthesis. Observations of the transformation of how catalysts are formed, especially from the morphological perspective, are essential for the rational design of the catalyst materials.

**Figure 15:**
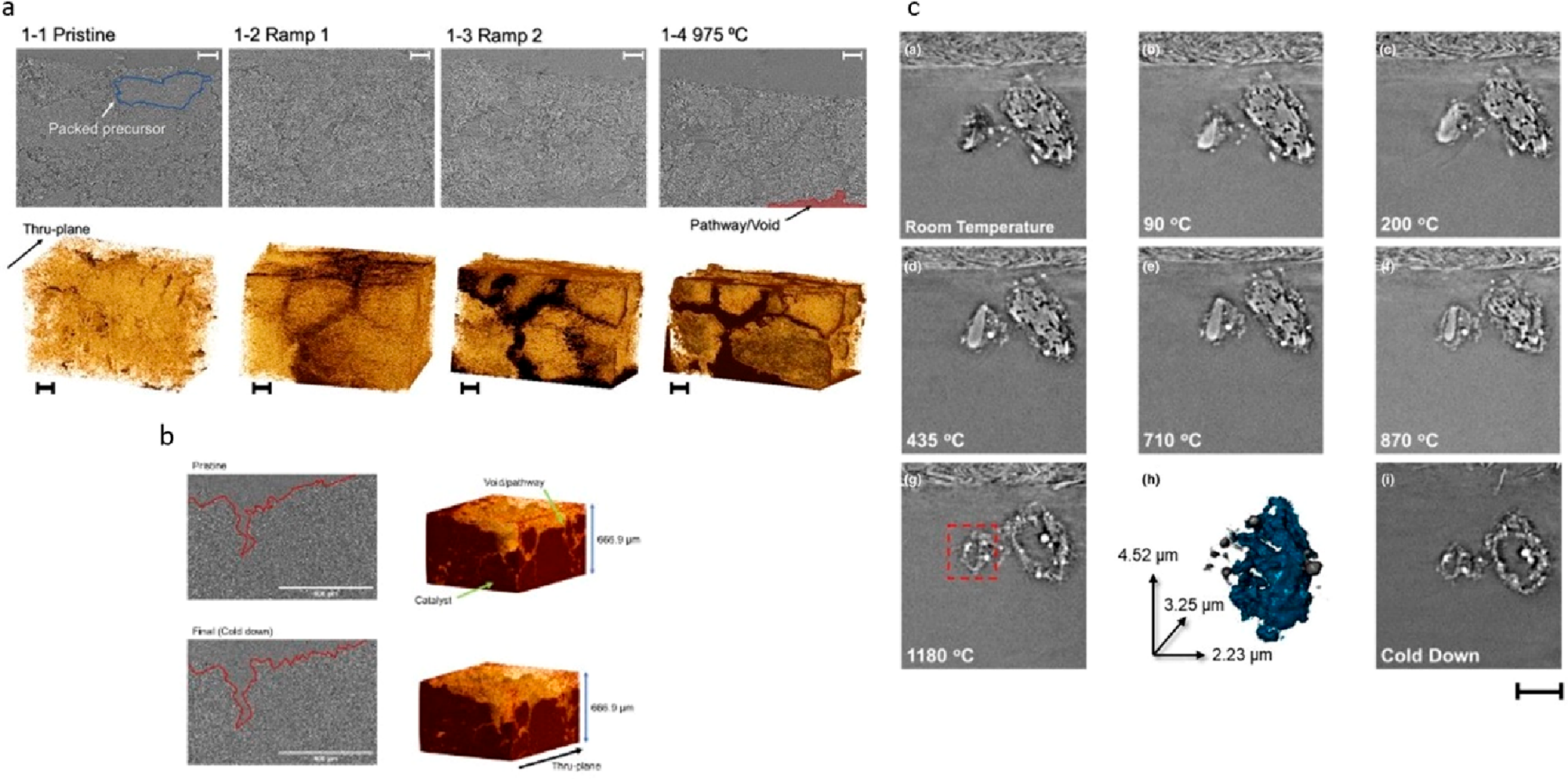
In situ X-ray CT data during pyrolysis leading to M–N–C catalysts. (a) micro-CT showing the transformation from the precursors (mixture of nitrogen-containing charge transfer organic salt, transition iron salt, and amorphous silica powder) to M–N–C materials during pyrolysis. Scale bar: 100 μm. Adapted with permission from ref (154). Copyright 2021 Elsevier. (b) micro-CT showing the transformation from the M–N–C materials to the state-of-the-art catalysts during pyrolysis. Adapted with permission from ref (162). Copyright 2022 Elsevier. (c) nano-CT showing the transformation from the precursors (mixture of nitrogen-containing charge transfer organic salt, transition iron salt, and amorphous silica powder) to M–N–C materials during pyrolysis. Scale bar for cross-sectional images: 5 μm. Adapted with permission from ref (154). Copyright 2021 Elsevier.

The setups mentioned above were used to study the pyrolysis process from which precursors are transformed to metal–nitrogen–carbon (M–N–C) catalysts via in situ micro-CT and nano-CT. The micro-CT provided transformation information on the catalysts as bulk materials. From the in situ data, the pathway formation and the porosity changes with the temperature increasing can be monitored as seen in Figure 15a. With the 3D information provided by the micro-CT, it can be observed that the porosity of the catalysts increased when the temperature was ramped up from room temperature to 975 °C. This phenomenon was consistent with the analysis using other characterization techniques, showing the evaporation of the nicarbazin inside the precursors. Also, the gas pathway formation was monitored when the samples were inserted into the furnace at 525 °C and released a large number of decomposed products. The same experiment was done to study the repyrolysis process, which retreated the formed M–N–C catalysts after it was acid-etched to improve their catalytic performance (Figure 15b).^162^ No noticeable change with the existing pathways was observed, although some trivial morphological changes were tracked. This indicates that the carbonaceous matrix of the materials was stable, which agrees with the other characterization results.

Nano-CT provides 3D information on the nanoscale. As already discussed, the additional optical components added to the beamline setup enable a much higher resolution for imaging. Ex situ nano-CT can provide the void distribution or micro porosity at nanoscale.^163^ Also, the phase ring allows the images to be demonstrated in the phase contrast mode. This provides a much clearer edge and a larger intensity difference between the two phases. Thus, element distribution analysis within the nanoscale is possible if there are only limited elements existing, and the atomic number differences between the elements are large. For M–N–C, the metal species can be identified easily from the nitrogen and carbon.^164^ As shown in Figure 15c, nano-CT was used to study the aged Fe–N–C catalyst.^154^ The iron oxides were segmented out from the nano-CT images, and thus, the fact that most of the metal oxides were distributed on the surface was revealed. The particle size distribution of the iron oxides was also provided, and it was observed that more than 47% of the metal species were in the range of 0.5–1 μm. The in situ nano-CT enables the tracking of specific particles during the pyrolysis. The experiment was done to understand how nicarbazin-based precursors formed catalysts.^154^ It was found that the particles shrunk at the beginning of the pyrolysis, and later a gap between the internal amorphous precursor phase and the external shell appeared. At last, only the external shell remained. This observation was direct proof that the transformation of amorphous carbon began at the edge of the two phases.

#### 4.2.3 Combination with Other Techniques

Most catalysis research, including catalytic synthesis, requires chemical information along with morphological observation. X-ray CT can be combined with the other X-ray techniques to gather additional information, not limited to morphological only.

XANES analysis has become more available at various X-ray CT beamlines, enabling simultaneous chemical mapping and morphological quantification. XANES can reveal the oxidation state of the active element of the catalysts. XANES-CT collected data as sinograms, which yielded 3D XANES mappings. In the XANES-CT data, one pixel is one XANES spectrum. The resolution is usually determined by the focal beam, which is in the microscale (depending on the beamline). XANES-CT is mostly done with a synchrotron beamline since it requires a large available energy range. Researchers in the battery field usually use in situ XANES-CT to observe the changes of oxidation state during the battery cycling.^165,166^ For catalysts, researchers used this technique to gain a chemical mapping, revealing the distribution of the active element oxidation states.^22^ X-ray fluorescence can also be detected along with CT. This enables a full observation of the catalyst materials from both morphological and chemical perspectives.^22^

### 4.3 Operando Studies

Operando studies are performed to gather performance data and structural images from X-ray CT.^28^ Beamlines can have stages so that electrochemical tests can be carried out while the sample is also imaged through X-ray CT. Operando studies can allow for real-time tracking of catalyst particles, ionomers, and water or gas migration inside the ROI.^38^ Operando tests can show fluid flow through porous media, catalyst performance, and material degradation. For fuel cells, the electrodes and the MEA can be investigated. For water electrolysis, PTLs can be studied through operando tests. For CO_2_ reduction electrolyzers, the GDEs can be the focus for operando studies. For redox flow batteries, the porous carbon felt electrodes and electrolyte flow can be investigated. For all of these, fluid flow can be viewed, while the cells are in operation.

#### 4.3.1 Fuel Cell Studies

Polymer electrolyte fuel cells (PEFCs) are a promising energy-conversion technology that operate at low temperatures, and they are primarily used for the transportation sector and power generation. To achieve their broad deployment, a reduction in cost and an improvement in durability are needed. Catalyst layers are critical components that use a platinum (Pt) electrocatalyst as the oxygen reduction reaction (ORR) catalyst. A current challenge is to reduce the amount of Pt used in the catalyst layer. Catalyst durability is a major obstacle to reduction of the catalyst loading, as currently the PEFC stacks are overdesigned to include more Pt for the end-of-life operation.^167,168^ X-ray CT is a valuable tool to predict the catalyst layer morphological changes during operation to pinpoint degradation phenomena. Water is a byproduct of ORR, and liquid water condensation in the catalyst layer and GDLs can lead to flooding that will block transport of the oxygen reactant to the catalyst layer. Therefore, understanding water management of PEFCs is critical to the design of catalyst layers and GDLs. Micro and nano-CT can provide valuable information on water distribution in the pores and correlate water distribution to the local wettability and performance of the PEFCs.

**Figure 16:**
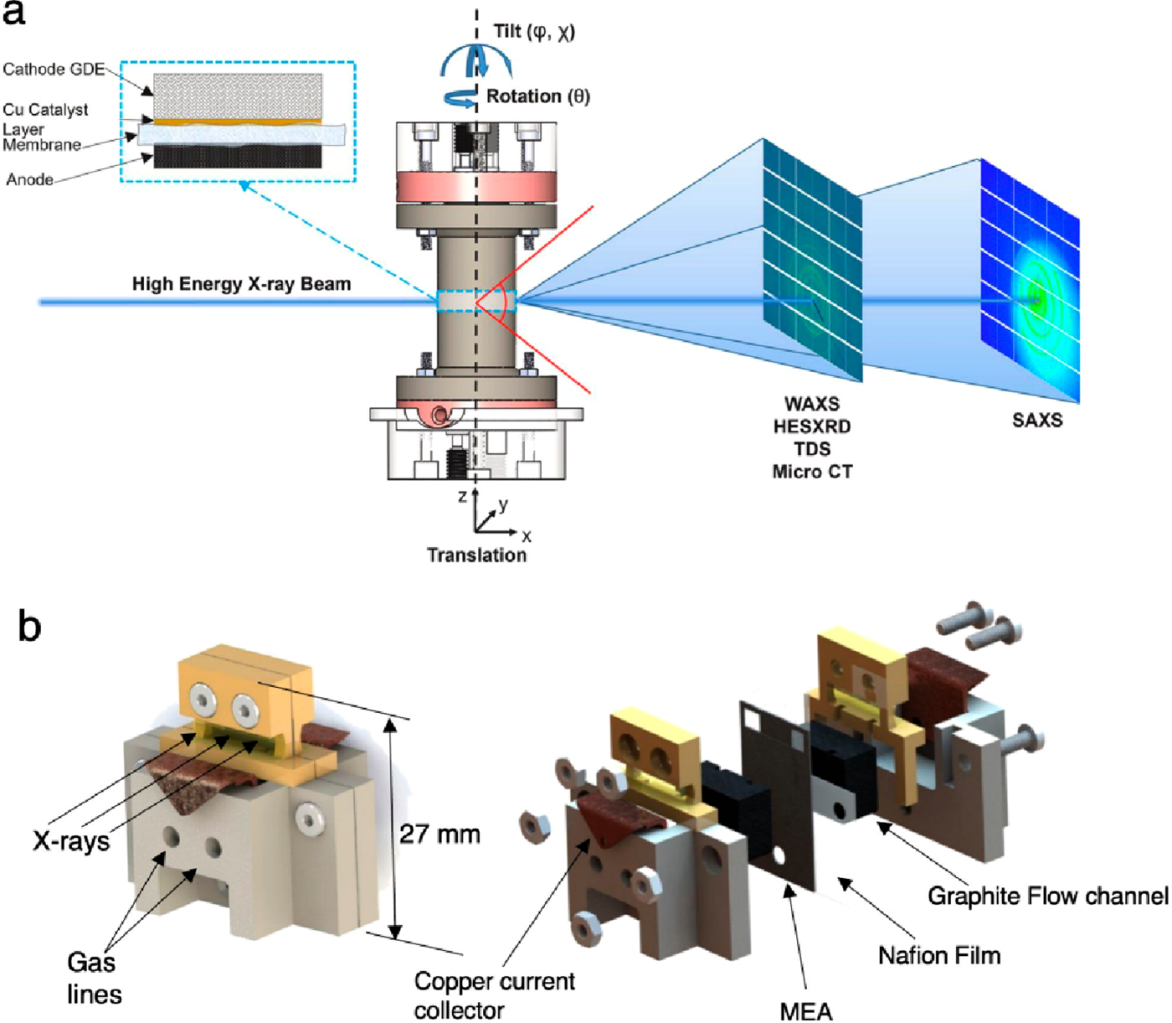
Examples of (a) micro-X-ray CT operando sample holder for PEFCs. Adapted with permission from ref (169). Copyright 2023 Elsevier. (b) nano-X-ray CT operando sample holder for PEFC. Adapted with permission from ref (17). Copyright 2020 IOP Publishing.

Both micro- and nano X-ray CT have been applied to study transport, reactivity, and degradation of fuel cells. Operando nano X-ray CT experiments are much rarer for PEFCs because of the challenges of experimental design and beam damage to polymer membranes. Typical sample holders for operando experiments are shown in Figure 16, where structural stability, X-ray transparency, mechanical compression, delivery of gases, and low contact resistances must be maintained. More details on operando cell design criteria can be found in Kulkarni et al.^12^

**Figure 17:**
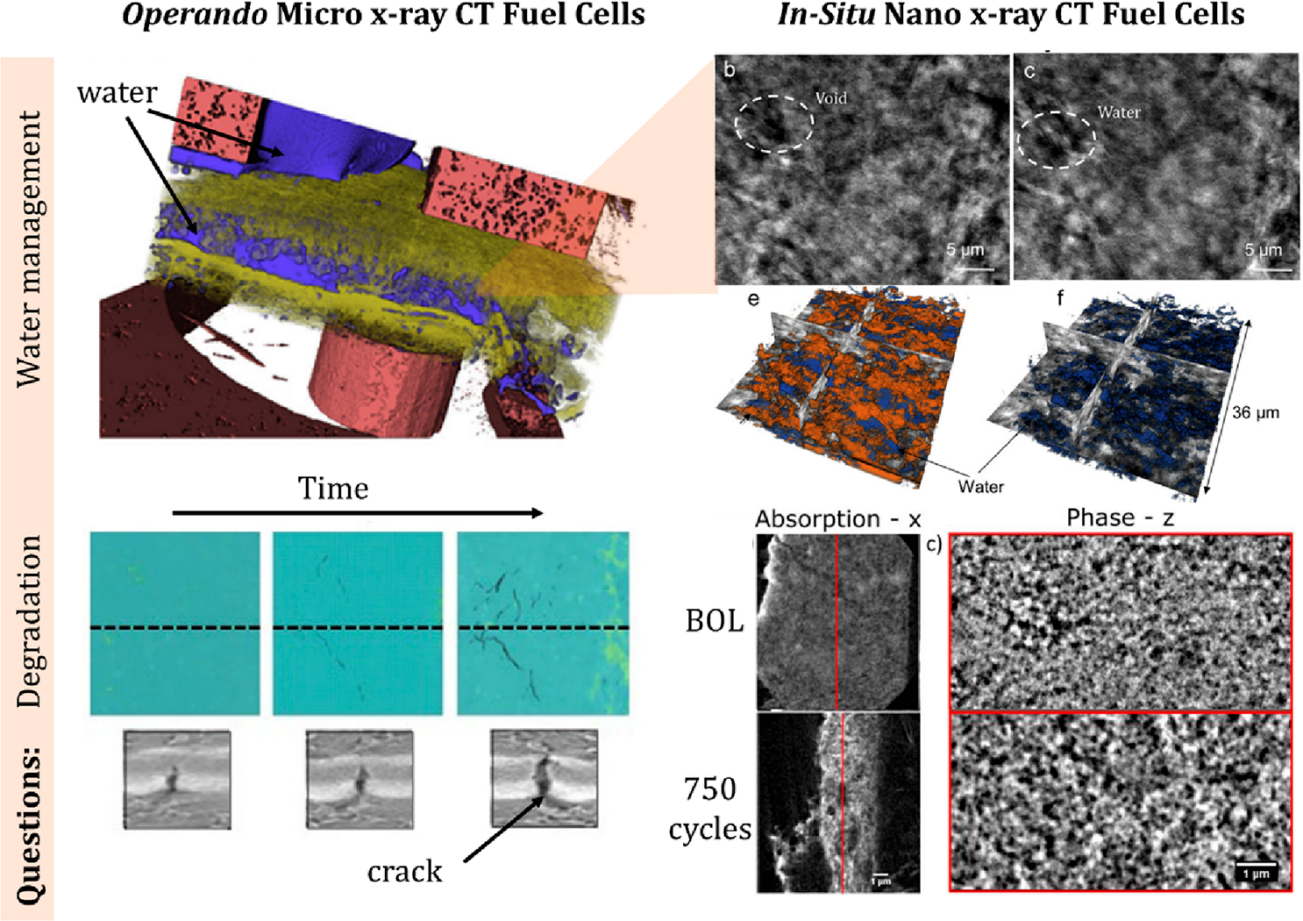
Examples of operando micro-X-ray CT studies and in situ nano-X-ray CT studies focusing on water management and catalyst or membrane degradation. Top images show water distribution in PGM-free catalyst layers using both micro and nano-CT. Adapted with permission from ref (29). Copyright 2018 Elsevier. The bottom left image shows the crack propagation through the MEA during accelerated stress test. Adapted with permission from ref (180). Copyright 2020 Elsevier. Bottom right shows catalyst layer thinning and structure changes during carbon corrosion AST protocol. Adapted with permission from ref (193). Copyright 2019 IOP Publishing.

Operando micro-X-ray CT studies for PEFCs primarily focused on two areas: (i) understanding water management and (ii) understanding material degradation during PEFC operation, as shown by Figure 17. For water management, the studies aim to understand primarily water distribution in the GDLs under various operating conditions and current densities or when materials are modified. Because micro-X-ray CT is limited to about 1 μm resolution, water distribution in conventional catalyst layers is not possible to probe. Recent studies have used radiography to describe water distribution in microporous layers (MPLs) that also have morphological features below the X-ray CT resolution.^170^ In situ X-ray CT was carried out on MPLs when water vapor was introduced, and images before and after the introduction of vapor were subtracted to show water distribution with differential images.^170^ The findings suggest that water drained to both sides of the MPL in the through-thickness direction, indicating that there is no preferential distribution of water to one side of the MPL. Furthermore, steady-state was achieved within 6 min of operation, indicating that water equilibration is not a fast process.

Water management in PGM-free catalyst layers that are orders of magnitude thicker (about 50–200 μm) compared to conventional catalyst layers has been studied by several works. These studies show that electrode morphology, wettability, and the morphology of the interfaces control water distribution in the catalyst layers.^171^ Peng et al.^117^ used operando X-ray CT to visualize water flooding in catalyst layers of alkaline exchange membrane fuel cells (AEMFCs). These catalyst layers are, by design, integrated into the GDLs. Therefore, their thickness is larger (∼50 μm) than that of the conventional catalyst layers. By adding PTFE to the catalyst layer ink formulation, they showed improved performance and no water flooding. The work also correlated results of neutron imaging that showed water distribution with lower resolution but a larger cell and X-ray CT, where higher pore-scale resolution was achieved. This work points to the importance of tailoring the wettability of the catalyst layer to its function; by adding PTFE, the catalyst layer became hydrophobic, ejecting more water, and preventing local flooding. Water distribution in the GDLs has been studied using operando studies extensively over the past decade, and the findings are well-summarized in ref (172). Recently, more studies have focused on material modifications to either change wettability or introduce perforations into the GDL or MPL to direct water transport.^173−175^ Here we will mostly focus on the most recent studies that are dynamic, as described later.

PEFC durability has been studied with X-ray CT, focusing on the following components: (i) membrane durability^176−181^ and (ii) catalyst cracking. Chen et al. in series of two articles^176^ explored the membrane and MEA failure due to edge failure under combined chemical and mechanical membrane degradation accelerated stress tests. Stoll et al.^182^ used operando micro X-ray CT to observe catalyst layer thinning during a start-up/shut-down accelerated stress test (AST), showing that larger carbon loss is observed for catalyst layers under the land locations compared to channels. In another study, the authors monitored crack formation at the inlet and outlet of a PEFC during carbon corrosion cycle.^115^ They found that the catalyst layer degraded more under the channels compared to the under the lands.

Dynamic studies are valuable, as they can show mostly transient water transport. With subsecond scans, TOMCAT at PSI is the only beamline currently that can carry out operando fuel cell experiments at this very high temporal resolution.^183^ For subsecond X-ray CT scans, several conditions are needed: (i) high X-ray flux to reduce noise, (ii) synchronized stage motion and slip-ring for tubing management, and (iii) minimization of beam damage at these high fluxes. Several important studies were carried out by the Büchi and Eller. Xu et al.^184^ compared water distribution in GDLs for two PEFC operating temperatures: 40 and 80 °C. They showed that phase-change induced flow is more dominant transport phenomena for 80 °C within the first seconds of current ramp to 1 A/cm^2^, whereas capillary fingering is the more dominant mechanism for operation at 40 °C. In another study, they compared transient and steady-state water distribution of operating PEFC with three different commercial GDLs.^185^ The same groups from PSI studied cold-start or operating fuel cells below freezing temperatures. PEFCs should be able to start at subzero temperatures. Sabharwal et al.^186,187^ used subsecond X-ray CT to understand dynamics of freeze starts from −30 °C using varied gas humidity. They showed that the GDL did not play a significant role in subzero startup, and most of the water produced was limited to the membrane and catalyst layer.

While X-ray CT at subsecond scan resolution is only available at TOMCAT, several studies use transient radiography to observe water thickness inside the operating fuel cells. Kato et al.^188^ used transient X-ray radiography (1.5 s resolution) to show water distribution in PEFC under different relative humidity (RH) and temperatures (*T*). They have shown that depending on *T* and RH, water transport can be either in liquid form, vapor form, mixed liquid–vapor, or only condensation near the ribs. Liquid water was observed under the lands but not in the channel. This discontinuity was difficult to explain due to the 2D nature of the radiography studies.

Thus far, we have focused only on PEFCs that operated below 100 °C. X-ray CT is also useful for high-temperature (HT) PEFCs, where the membrane is typically acid doped with phosphoric acid, and phosphoric acid leaching is a major degradation issue for these HT-PEFCs. Studies have studied phosphoric acid leaching^189^ and water distribution within HT-PEFCs, indicating the importance of the MPL design. Furthermore, in-house catalyst layer design with a minimum number of cracks was revealed to be desired to prevent acid leaching.^190^ Another study^191^ has looked into HT-PEFC degradation modes using X-ray CT, showing that under current cycling the phosphoric acid amount in GDL was unchanged but its distribution between the layers changed. Furthermore, membrane swelling and catalyst redistribution were observed too. Phosphoric acid leaching can be contained by engineering materials, such as a graphene interlayer between membrane and catalyst layer, as shown by Chen et al.^192^ Single-layer graphene increased phosphoric acid retention, blocked hydrogen crossover, and enabled operation at higher power densities.

In all of the studies reviewed here, operando cell design is critical for experiments because PEFCs for operando studies should be benchmarked against the PEFCs in the laboratory. The studies reviewed here used micro-CT for water management and degradation studies. Nano-CT operando PEFC studies are scarce and mostly involve ex situ imaging because in situ and operando imaging is still very challenging. Kulkarni et al.^12^ reported the design of an operando nano-X-ray CT operando cell that was operated at various synchrotron beamlines. Because of the lower energy of nano-X-ray CT beamlines (8 keV) and high flux, the membrane and ionomer get beam damaged. Furthermore, due to the high aspect ratio of the MEA, missing angles, and movement of the membrane during the scan, resolving the catalyst layer microstructure and water distribution is challenging. The use of K–B mirrors at the European Synchrotron Facility (ESRF) enables imaging at higher energies (17.5 keV), which will result in less beam damage and a possibility to see water within the catalyst layer, for example.

Lastly, combined multiscale and multimodality techniques were used to study PEFC degradation and water management. White et al.^193^ used a combination of operando micro- and ex situ nano-CT to understand crack formation, catalyst layer thinning (micro-CT), as shown by Figure 17, and nanoscale cracks and pore-size distribution changes (nano-CT). The nanoscale morphology of the catalyst layer was observed using Zernike phase contrast imaging. Normile et al.^29^ used operando micro X-ray CT for PGM-free electrodes to show water distribution and correlate it to interfacial and catalyst layer porosity, as shown by Figure 17. At the same time, an in situ nano-CT study was conducted to show water distribution in the catalyst layer at higher resolution.

#### 4.3.2 Electrolyzer Studies

Polymer electrolyte membrane water electrolysis (PEMWE) is a promising technology to produce green hydrogen at low temperature and high efficiency.^194,195^ As renewable energy penetration grows and the market shifts to a hydrogen-based economy, the demand for hydrogen turn-key solutions is increasing. One of the main barriers for PEM electrolysis deployment is cost, which is contributed mostly by expensive IrOx catalysts used on the anode for oxygen evolution reaction.^196^ Reducing Ir loadings and increasing their utilization are key for widespread commercialization of these systems. Along with reducing Ir loadings, optimization of critical components such as PTLs is essential for reducing overpotentials and increasing catalyst utilization.^197,198^ Previous attempts at understanding bulk oxygen transport through channels and PTLs involved using techniques like neutron imaging and optical microscopy,^199−203^ but they lack spatial resolution and 3D images cannot be obtained.

**Figure 18:**
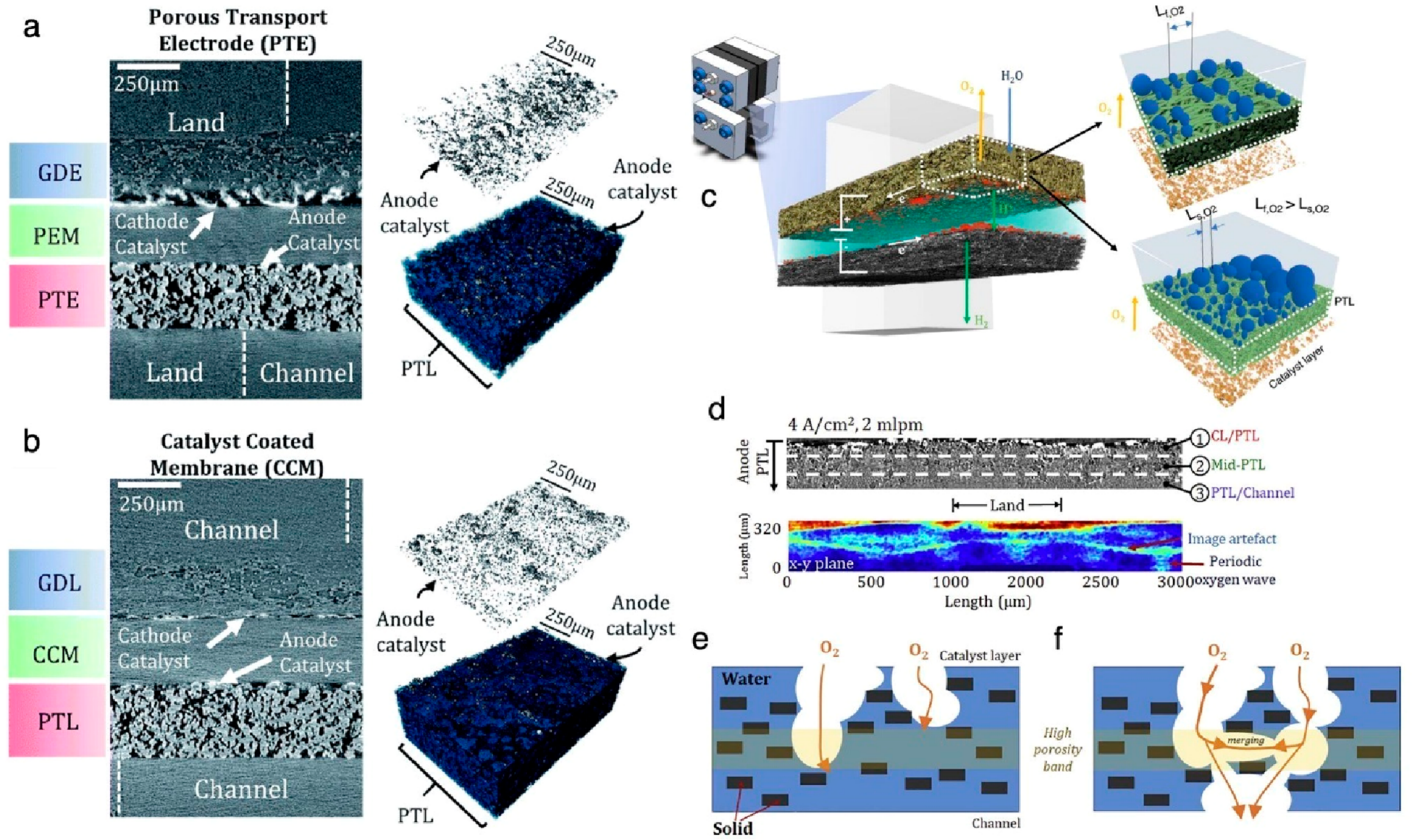
Examples of X-ray CT used for studying PEM electrolyzers. (a and b) 2D cross sections and corresponding 3D renderings of PTL and catalyst layer for two-electrode configurations, porous transport electrode (PTE) and catalyst coated membrane (CCM) respectively. Adapted with permission from ref (102). Copyright 2020 Royal Society of Chemistry. (c) 3D reconstructed view of operando cell used to quantify the triple phase contact area and gas flow in channels with varying PTL morphologies. Adapted with permission from ref (101). Copyright 2022 Elsevier. (d) 1–3 portions of the PTL that are located at the CL/PTL interface, middle of PTL, and PTL/channel interface. (Bottom) 2D oxygen content of the PTL at the *x*–*y* plane by using the Z-project method. (e and f) A conceptual schematic showing transport of oxygen in the PTL. Panels d–f adapted with permission from ref (87). Copyright 2020 iScience.

The use of X-ray CT for establishing relationships between the PTL microstructure and its effect on fluid transport to and from the anodic interface is gaining widespread popularity due to its versatility and quality of data obtained. Figure 18 shows examples of studies conducted with X-ray CT for studying microscopic and bulk transport processes in PEM electrolyzers. X-ray CT was initially used to study the structure–performance relationships between PTL structure and electrolyzer performance as an ex situ technique.^36,204^ Although ex situ investigations gave semi-empirical relationships between the PTL microstructure and electrolyzer performance, a more in-depth insight into anodic interfacial properties and gas transport is essential in order to enable component optimization and catalyst loading reduction. Operando X-ray CT experiments give the highest insight into these microscopic interfacial properties of relevance. The first operando X-ray CT experiments for electrolyzers were conducted by Leonard et al. to quantify the morphology evolution and oxygen bubble transport.^205^ Since then, several operando studies have been conducted by various groups with their own operando cells compliant with various beamlines. The general design guidelines for operando cells and their use for operating in fuel cell or electrolyzer mode have been reported by Kulkarni et al.^12^

Figure 18a,b shows the 2D cross-sectional view and corresponding 3D rendering of a PEM electrolyzer as observed with X-ray CT. The cathode and anode catalyst layers appear the brightest due to a higher Z-contrast produced by heavy elements like Pt and Ir, respectively. The Ti PTL has a higher contrast as compared to that of the cathode GDL made of carbon. These differences in contrast make it easier to segment the different phases individually. This can be used to quantify the geometric triple-phase interfacial contact between the catalyst, PTL, and the membrane and estimate the catalyst utilization especially since electrochemically active surface area (ECSA) in electrolyzers cannot be accurately quantified with traditional hydrogen adsorption cyclic voltammograms in full cells.^206,207^ Recent studies have focused on understanding the factors that affect catalyst utilization and overpotentials by interfacial analysis.

Schuler et al. showed that the catalyst utilization is directly affected by the PTL surface morphology and kinetic and ohmic overpotentials are directly related to increased catalyst utilization.^198,208^ Kulkarni et al. studied the interfacial properties of two different electrode configurations.^101^ The first configuration is the catalyst coated membrane (CCM), where the catalyst may be deposited on the membrane. The second configuration is the GDE, where the catalyst may be deposited directly onto the PTL. For the GDE configuration, a commercial fiber PTL and sintered PTL were used. Figure 18c shows the complete 3D volume rendering of the PEMWE cell used in the study. Their results showed direct correlations between the interfacial contact and its influence on all cell overpotentials and steady-state durability. They concluded that CCM configurations with low porosity sintered PTLs can enable high interfacial contact and reduce mass transport overpotentials at high current density as corroborated with their X-ray radiography results, quantifying the oxygen removal into the channels.

Although oxygen transport into the channels can be quantified with high-speed X-ray radiography, its transport through PTLs cannot be observed directly because PTL produces significantly higher contrast than oxygen, making it difficult to segment the oxygen phase from Ti. Lee et al. used ex situ X-ray CT to characterize PTL morphology and observed oxygen transport through PTLs using a microfluidic platform under optical microscopy.^209^ Their results showed capillary fingering as the dominant transport regime and that PTLs can be modified to have limiting pore throats closer to the interface to reduce mass transport overpotentials.

Following that, Satjaritanun et al. used acid-treated carbon GDLs to imitate fiber PTLs in operando X-ray CT studies to directly observe oxygen transport through the PTL.^123^ They observed oxygen taking preferential pathways for removal and observed a spatial periodicity of ∼400 μm mostly influenced by PTL pore size distribution, as shown in Figure 18d–f. Perhaps the most successful study to observe oxygen distribution through actual PTLs was done by De Angelis et al. by using stained water in operando cells.^124^ The water was stained with 5% iodic acid to increase its contrast against PTLs, which made it possible for segmentation using novel image processing techniques. Figure 18d,e represents the 3D rendering of the fiber PTL with oxygen phase and oxygen transport pathways, respectively. The overall oxygen content in the PTL increases with increase in current density. However, the overall oxygen saturation decreases from the catalyst layer to the channel as represented in Figure 18f,g, respectively. From the findings, they proposed the idea of anisotropic PTLs where the in-plane pore throat size distribution is smaller than the through plane to ensure efficient water transport to the catalyst layer and oxygen removal. These findings also strongly point to the direction of using MPLs to increase interfacial contact and reduce overpotentials.^210^ Hence, future X-ray studies for PEM electrolysis are directed toward the optimization of MPLs and durability.

#### 4.3.3 CO_2_ Reduction Reaction Studies

**Figure 19:**
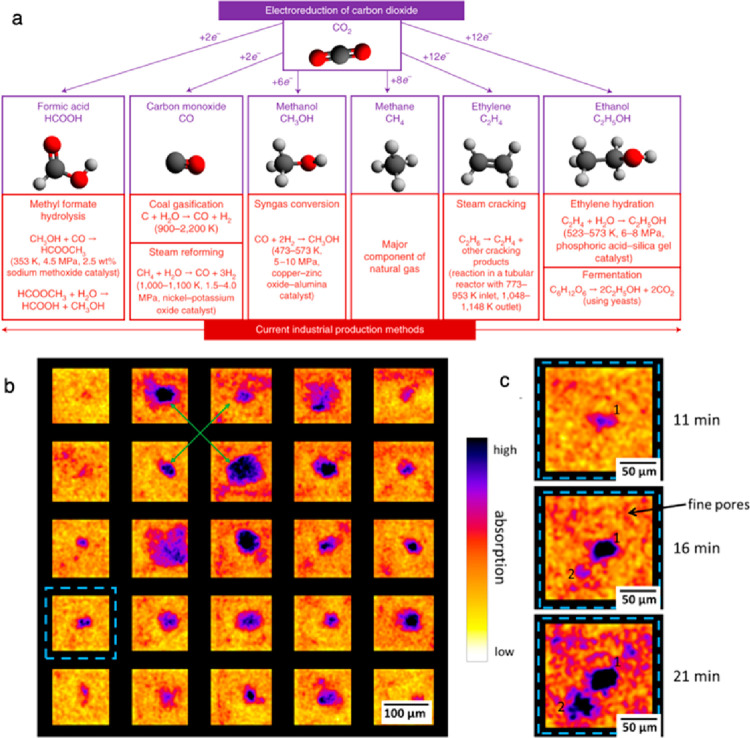
(a) Current large-scale methods to produce various industrial feedstock chemicals mostly produced by fossil fuels. CO_2_RR can be an alternative to offset these fossil fuel-based methods. Adapted with permission from ref (214). Copyright 2019 Springer Nature. (b) Operando synchrotron imaging of the electrolyte distribution within a CO_2_RR GDE with 92 wt % Ag at 0.2 V, radiography of the GDE, Ni mesh (recalculated, black), pores filled with electrolyte (green arrows), and (c) enlargement of the pore system (blue dashed rectangle in panel b) between the mesh wires. Adapted with permission from ref (215). Copyright 2022 The Electrochemical Society.

Capturing carbon dioxide from the atmosphere or from industrial flue gases is essential for environmental remediation and creating carbon negative or carbon neutral industrial processes. The electroreduction of carbon dioxide (CO_2_RR) to value-added products presents a strong promise for offsetting industrial feedstock gases that would typically be obtained from fossil fuels such as methane, carbon monoxide, ethylene, formate, etc.^211,212^ The electrocatalytic reduction of CO_2_ however presents major technological challenges since CO_2_ is a thermodynamically stable molecule and requires crossing of a high free energy barrier for splitting.^213^ CO_2_RR is typically performed with anodic water oxidation (oxygen evolution reaction) mainly because of advances in OER catalysis and membranes from the field of PEM/AEM water electrolysis and can therefore serve as a quasi-reference electrode.^214^ The cathodic CO_2_RR at a heterogeneous catalyst surface can produce a number of different products, as depicted in Figure 19a. These products have a significant overlap between standard reduction potentials along with competing with hydrogen evolution reaction (HER) that happens at 0.0 V vs RHE. Hence, selectivity is a major challenge in these systems.

Hori et al. in 1989 were the first to show the efficacy of silver as a good catalyst for CO_2_RR since it showed over 80% conversion of CO_2_ to CO.^216^ Additionally, they also showed that a breadth of products including methane and ethylene can be synthesized simultaneously in a single-step electrochemical reaction using copper as catalyst.^217,218^ Since then, several studies have reported increasing selectivity and activity of Ag and Cu based catalysts such as using Ag nanoparticles with ionic liquids^219^ and nanoporous Ag catalyst^220^ reaching CO Faradaic efficiencies of >90%. Hence, CO_2_RR electrolyzers operating at an industrially relevant current density of over 200 mA/cm^2^ have become possible with both Ag and Cu based electrocatalysts.^221−224^ However, although catalyst development is a major component, optimizing electrolyzer design is essential for reducing overpotentials and achieving economically viable scales. Janáky and co-workers have pioneered design of vapor fed continuous flow cells and stacks including zero gap cells for CO_2_RR with faradaic efficiencies of over 95%.^225,226^ For these vapor-fed systems, GDEs are often the electrodes of choice that employ porous catalyst and diffusion media similar to fuel cells that allow high current density operation.^227^ GDEs however have a major challenge of flooding that disrupts flow of CO_2_ to the catalyst layer and causes reduction in selectivity toward CO_2_RR products.^228,229^ Flooding of water in GDLs has been well studied for fuel cells, but in the case of CO_2_RR, flooding issues are much more complex since it occurs through various interrelated processes and the high negative potentials affect the wettability of the GDL fibers.^230^ Hence, there is an immense scientific opportunity to understand fundamental and multiscale processes in GDEs with X-ray CT and other operando X-ray techniques. Various ex situ and operando studies were conducted on silver-based GDEs coupled with ORR to understand the effects of microstructure and electrolyte transport through these electrodes.^231−235^ Paulisch et al.^232^ made seminal contributions to utilizing X-ray CT for understanding electrolyte distribution in silver electrodes used for chlor-alkali electrolyzers and, later, by extension to CO_2_RR electrolyzers. Lee et al.^236^ first used operando X-ray CT to visualize gas bubble formation in the electrolyte chamber in CO_2_RR cells. They explained how the accumulation of CO_2_ and syngas bubbles at the PEM and GDE interface region affected cell voltage at various current densities. Since the OER is still an anodic reaction of choice for CO_2_RR electrolyzers, the insights on the complex multiphase flow in PTLs from PEM electrolyzers are highly relevant in this context as well. Hoffman et al.^215^ later developed operando cell for monitoring electrolyte distribution and bubble formation during ORR and CO_2_RR and showed crystallization effect of electrolyte caused by the hydrophobic nature of the GDE. They were also able to map electrolyte distribution and visualize blockage of GDE surface by HER bubble formation as shown in Figure 19b,c. These studies are important for designing highly efficient GDEs and optimizing the transport of reactants at reaction interface areas. Along with X-ray CT imaging, techniques such as synchrotron XANES can be employed simultaneously to understand oxidation states and the chemical nature of catalysts in operando conditions.

#### 4.3.4 Redox Flow Battery Studies

**Figure 20:**
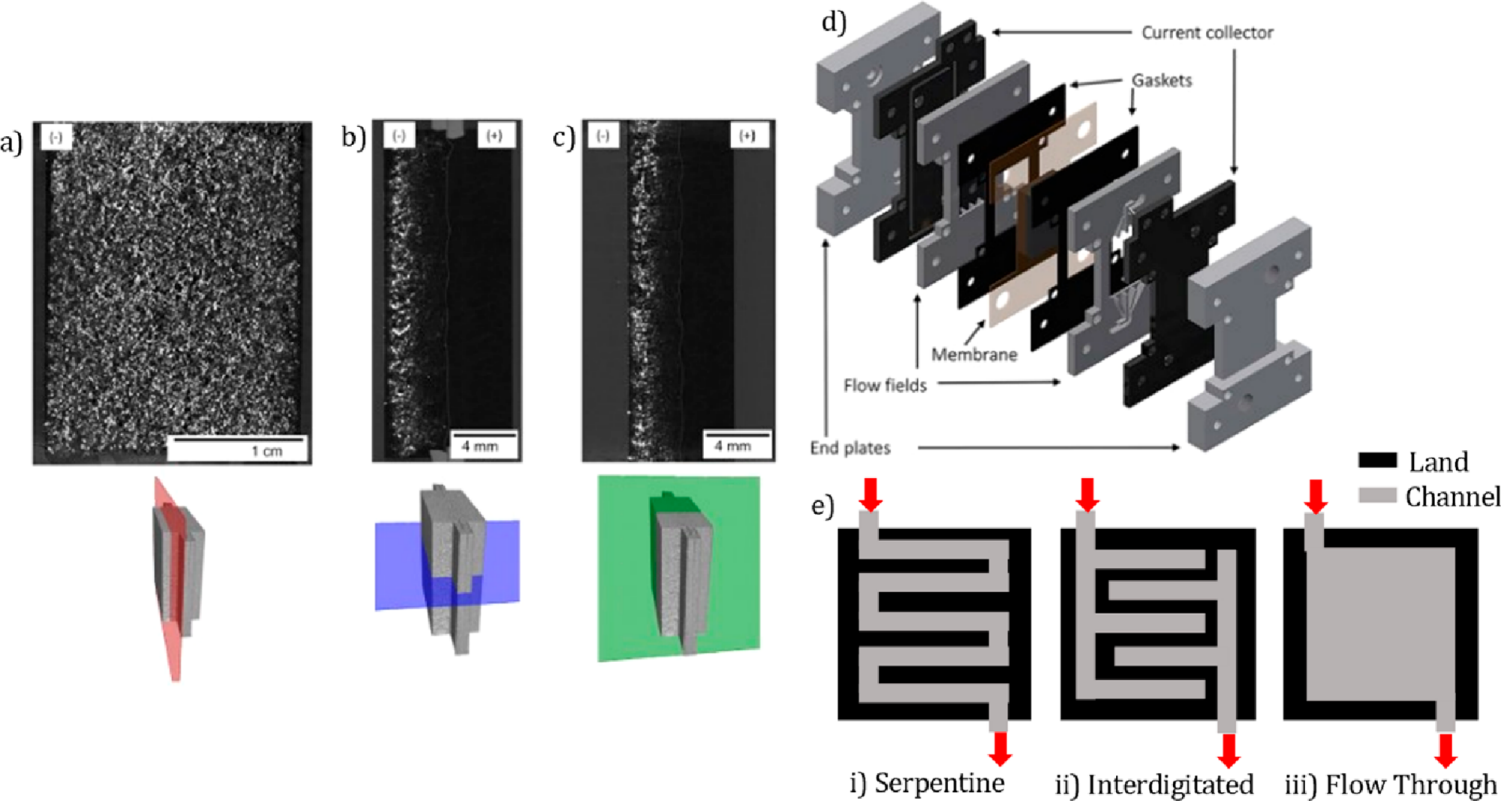
(a–c) Recreation of the dry version of the cell from X-ray CT images on each plane, along with (d) the operando cell used to gather the images. Adapted with permission from ref (33). Copyright 2020 Elsevier. (e) Examples of different flow field geometries that can be applied to the redox flow battery cells, and these are mentioned in the X-ray CT study by ref (34).

Redox flow batteries are a simple yet durable type of energy storage system that utilize reduction and oxidation reactions to store and release energy.^237−241^ The electrodes used for these devices are made of a porous carbon structure that can undergo structural changes throughout compression and operation.^242^ The microstructure of the electrodes affects mass transport, pressure drop, and flow characteristics.^243^ The porous electrodes also have a large effect on the power density, overpotentials, and losses associated with battery performance, so it is important to characterize the electrode structure at the micron level through operando X-ray CT studies to understand how the electrodes function and see how to improve upon the electrode design.^240,244−246^ These scans can show changes in the porosity, pore size distribution, and tortuosity within the porous electrodes as the operando cell operates. The flow field is another factor to consider for the overall cell design and functionality. Common designs include serpentine, flow-through, and interdigitated and are shown in Figure 20e. Special cell designs are necessary to allow for X-ray imaging of the electrodes to be completed while in operando conditions.^33,34^ The area of interest, the porous electrodes in this case, needs to be available to receive the X-rays emitted from the synchrotron in order for the images to be collected. The combination of X-ray CT and cell testing can show catalyst and electrode performance and electrolyte flow while characterizing the structure of the electrode. This section will review operando studies into redox flow battery electrode performance and electrolyte flow.

Catalyst and electrode performance can be investigated by operando X-ray CT tests. A study by Gebhard et al.^33^ used X-ray CT and X-ray radiography to investigate the stability and performance of bismuth-modified carbon felt electrodes compared to unmodified carbon felt electrodes in a vanadium redox flow battery operando cell. The operando cell design along with X-ray CT recreations of the dry version of the cell are shown in Figure 20a–d. Both electrodes’ performance and structures were characterized through in operando X-ray computed tomography scans and then compared against each other.^33^ Bismuth has a higher level of absorption of X-ray radiation than the normal carbon felt electrode, so it showed a much darker gray color in the scans.^33^ The image analysis showed that the bismuth would be deposited on the electrode as the cell charged with most of the deposition occurring closer to the current collector at the entrance of the cell.^33^ X-ray CT can also be used to track electrode degradation throughout battery operation.^247^ Scans can be taken at different times of continuous battery operation to visualize how the porous electrodes change as the battery operates. A study from Trogadas et al.^247^ used micro-X-ray CT to monitor a couple important changes to the electrodes: electrode fiber agglomeration and electrochemical oxidation of the carbon. Analyzing degradation mechanisms in the electrodes will help inform the design of both carbon felt electrodes and modified electrodes as seen in the study by Gebhard et al.^33^

**Figure 21:**
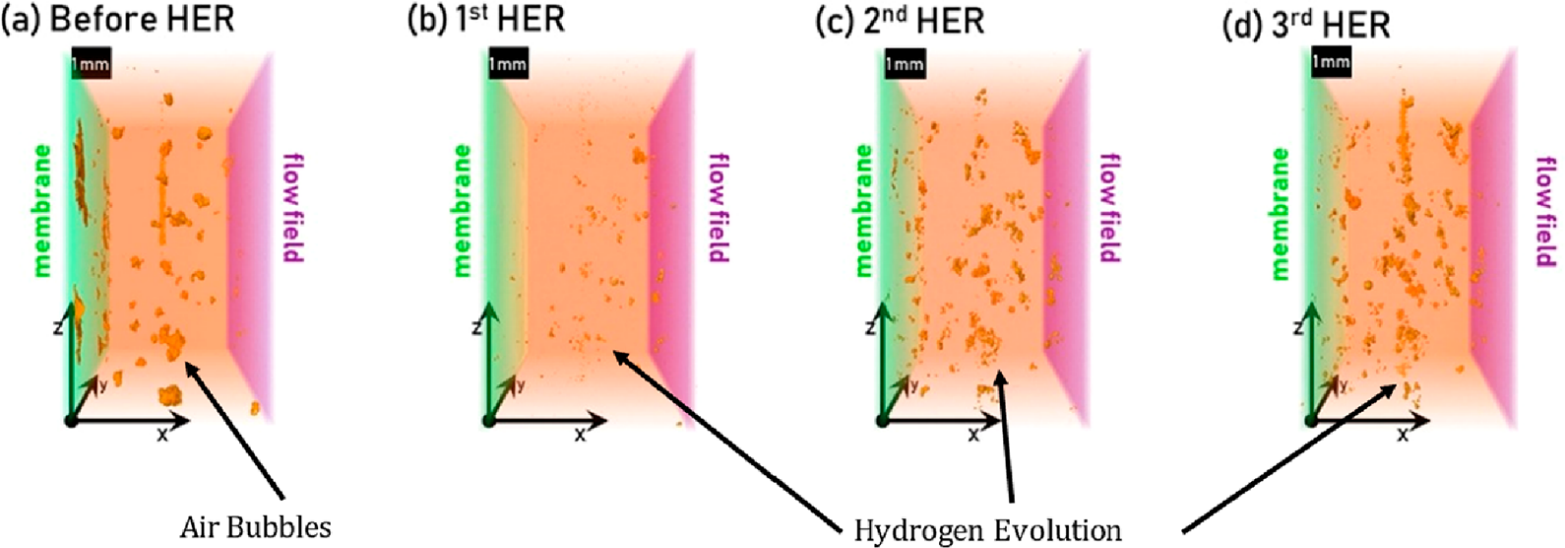
(a) Air remaining inside the porous electrode; (b and d) bubble evolution after subsequent periods of undergoing Hydrogen evolution reaction conditions. The shown channels are reconstructed after X-ray CT imaging. Adapted with permission from ref (34). Copyright 2020 John Wiley and Sons.

Another aspect that can be investigated with X-ray CT is electrolyte flow through the porous redox flow battery electrodes. A study by Bevilacqua et al.^25^ looked into the effects of electrolyte and electrode compression on flow using X-ray CT. After segmenting the images taken, the liquid distribution in the carbon felt electrode was able to be extracted.^25^ The results were able to show the effect of different electrolyte and electrode activation versus nonactivation on the wetted surface area inside the electrode.^25^ It was determined that introducing surface groups during thermal activation led to a higher saturation and a higher electrochemically active surface area inside the electrode.^25^ Following further with electrolyte flow studies, a paper by Eifert et al.^34^ investigated the effect of different flow field geometries on the electrolyte flow dynamics. The flow designs that were tested were serpentine, interdigitated, and flow-through.^34^ The flow-through design achieved the highest electrode saturation with an electrolyte injection method, but the serpentine design had the highest electrode saturation with continuous electrolyte flow conditions.^34^ It was possible to look for remaining air in the porous electrodes and evidence of parasitic side reactions while investigating the electrolyte flow using X-ray CT. To do this, hydrogen evolution bubbles had to be tracked. The side reaction was tracked by its current response and decrease in saturation, and the bubbles were visualized through X-ray CT and are shown in Figure 21.^34^

A study by Köble et al.^35^ focused on the time and spatial resolved flow for the electrolyte inside redox flow batteries. This study sought to link electrode compression to electrode saturation and found that the higher the compression, the higher the saturation with above 50% compression giving 97% saturation.^35^ Synchrotron X-ray radiography was used to visualize the electrolyte invasion into the electrode.^35^ As the compression on the electrodes was increased, effects at the boundaries of the electrode that hindered electrolyte injection were able to be overcome, but the increasing compression caused a decrease in the diffusivity and permeability of the electrolyte in the electrode.^35^ 3D reconstructions of the electrodes from these types of experiments that investigate electrolyte flow characteristics can be applied to mathematical models of redox flow batteries (a closer look into modeling is described in the next section). These models can more easily investigate the mass transport and fluid mechanics inside the battery electrodes at different operating conditions.^237,244,245,248^

#### 4.3.5 Mathematical Modeling Using X-ray CT Imaging

X-ray CT data sets are useful for modeling, as they can provide geometry of computational domains. In situ/operando experiments can serve as model validation data sources. Typically, experiments are costly and can be time-consuming when looking at multiple samples or when extensive design of experiment is needed. Computational fluid dynamics (CFD) modeling can be utilized as a method to narrow down the samples of interest or to design extensive parametric sweep of operating conditions and geometry with minimal experimental effort. Using X-ray CT scans of different porous GDLs or PTLs, real 3D reconstructed geometries can be produced. Numerical modeling simulations run faster than experiments, are less costly, and can use the entire geometry generated by X-ray CT.

**Figure 22:**
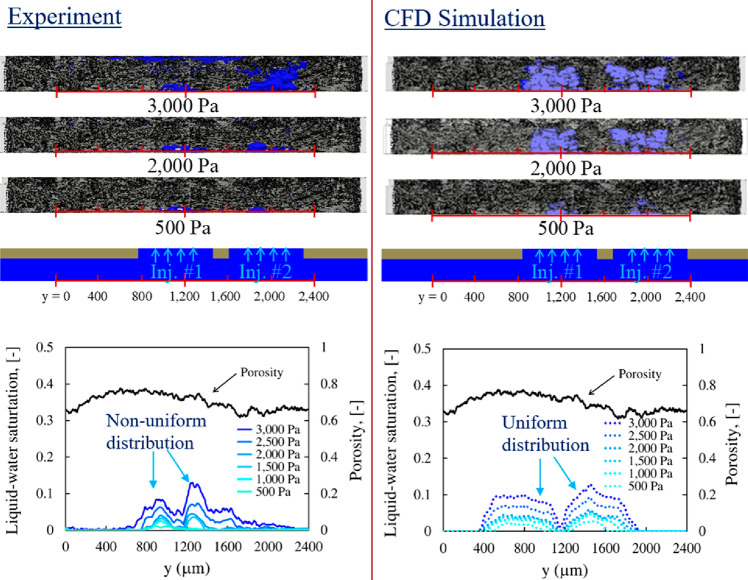
Experimental and simulated liquid-water saturation inside the compressed GDL with two injection holes in the bottom under the channel. (top-left) Cross-sectional volume of the liquid phase of experimental with the pressure of 500, 2,000, and 3,000 Pa. (top-right) Cross-sectional volume of the liquid phase of CFD simulation with the pressure of 500, 2,000, and 3,000 Pa. (bottom-left) Experimental liquid-water saturation profiles, and (bottom-right) simulated liquid-water saturation profiles. Adapted with permission from ref (249). Copyright 2018 IOP Publishing.

Satjaritanun et al.^111,249^ reconstructed GDL geometries from X-ray CT scans to predict liquid transport phenomena through porous media. GDLs are crucial to the operation of fuel cells, and a better understanding of liquid transport through the pore structure can aid in better fuel cell efficiency. In these works, CFD modeling is used to simulate liquid evolution, saturation, and solid–liquid interactions through different GDLs. First, it is necessary to produce a validated model to ensure that the predictions are accurate. XFlow, a commercial CFD solver that utilizes the Lattice Boltzmann Method (LBM), was chosen for these studies. The LBM is beneficial when simulating complex geometry because it uses particle-based tracking, which does not require meshing of the geometry. This means that the integrity of the sample structure is maintained. The model was used to predict the local saturation inside different samples. When compared to the experimental data, the model predicted saturation data comparable to experiments. This means that the numerical modeling technique using LBM is an acceptable way to simulate liquid transport through porous media. With a validated GDL model, the next task was to reproduce sample geometries with the addition of an MPL. An MPL is added to the GDL to aid in water management and gas removal. X-ray CT scans were used to reconstruct the sample geometries. Precise imaging is important for these samples, because the MPL has microscopic cracks on the surface. Liquid breakthrough pressures were first observed experimentally for different GDL/MPL samples. Then, XFlow software was used to predict breakthrough pressures through the same samples. When data were compared, the model agreed with the experimental results. Therefore, X-ray CT scans combined with the LBM can predict real liquid transport phenomena in porous media. With the model validated, the last task was to develop a model geometry that would emulate the internal environment of a fuel cell. To provide insight into the transport phenomena inside the GDL during fuel cell operation, a land-channel geometry was created to represent the inside of the fuel cell apparatus. This allows for the influence of the GDL geometry on liquid transport inside an operating cell to be observed. Single and multiple injection points were used to obtain predictions on liquid evolution and saturation behavior under the land and channel areas. Predicted water evolution through the GDL samples agreed with the visualized data produced from X-ray CT scans of experiments, shown in Figure 22. Results showed that the GDL geometry influences the liquid-water saturation and the breakthrough pressure.

**Figure 23:**
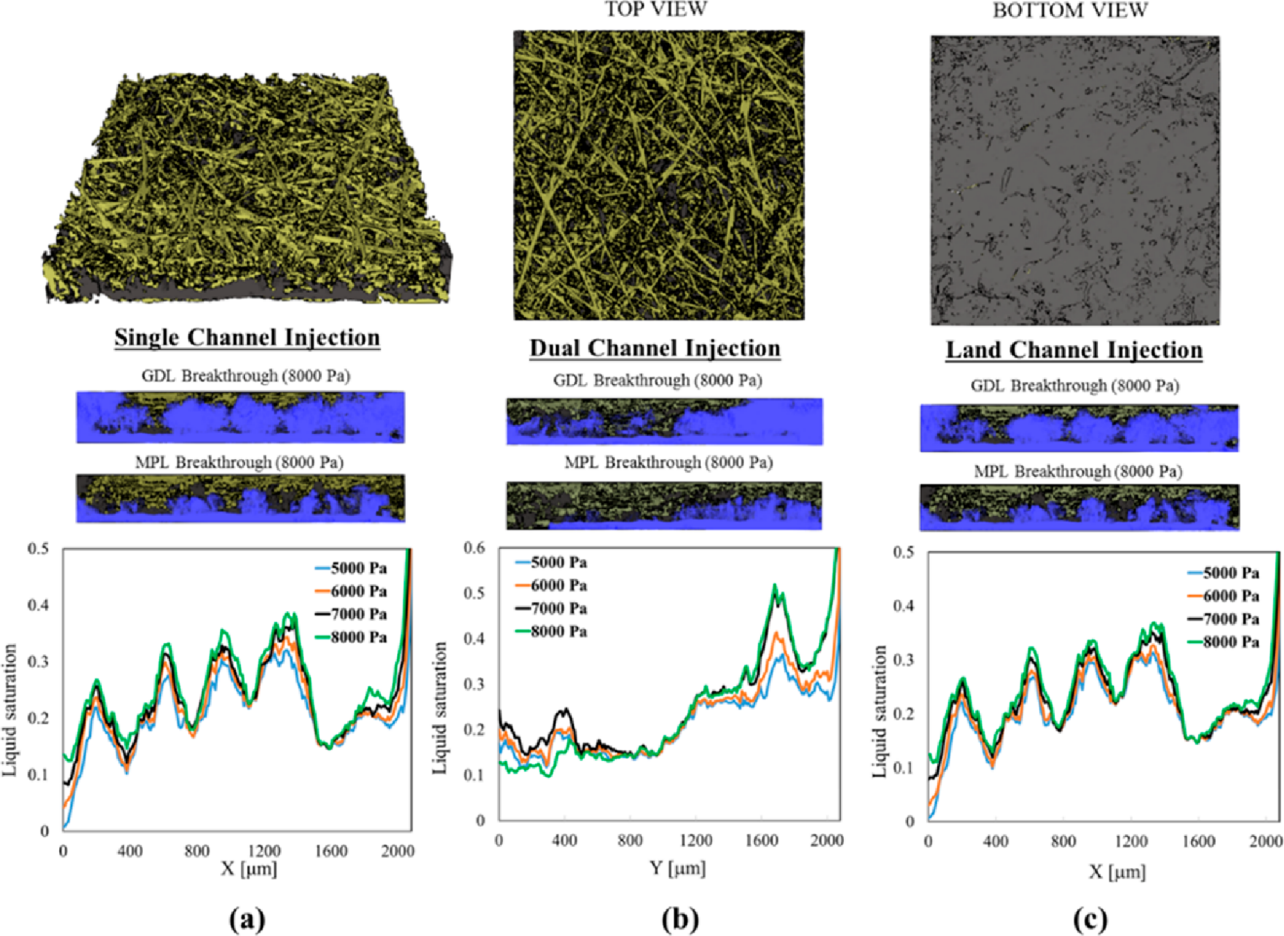
Liquid saturation inside sample SGL25BC in all three injection cases. Breakthrough at the MPL and GDL are shown for each case. (a) Single injection orientation. (b) Dual channel injection orientation. (c) Land channel injection orientation. Adapted with permission from ref (250). Copyright 2021 IOP Publishing.

Sepe et al.^250−252^ use the validated LBM model to simulate liquid transport through different gas diffusion layer samples. The objective of these studies is to observe the influence of the GDL and MPL geometry on liquid transport phenomena. A two-rib, one-channel geometry orientation was used to simulate the internal environment of a fuel cell. The first task was to predict liquid saturation and evolution in GDL samples without an MPL. Four commercial carbon fiber GDL structures were chosen for this study. Micro X-ray CT scans were used to reconstruct the 3D geometries of the four GDL samples. GDL geometries were imported into XFlow, which uses the LBM and does not require meshing of the geometries. It is necessary to create precise 3D reconstructed geometries to accurately predict liquid transport through the sample. Liquid injection points with varying inlet pressures were used to observe the influence of the GDL geometry on liquid transport phenomena. Results showed that the path the liquid follows to exit out of the channel is dependent on the GDL pore structure. Without X-ray CT scans of the GDL, prediction of liquid evolution would not be possible. The next task was to use micro-X-ray CT scans to generate GDL structures with the addition of an MPL. Proton exchange membrane fuel cells require a GDL to maintain saturation level and aid in the removal of gas from the catalyst surface. Often, MPL is added to the GDL surface to improve saturation. The LBM was applied to GDL samples with an MPL to study the influence that MPL has on saturation and liquid evolution. A two-rib, one-channel geometry with different injection points was used to simulate liquid transport inside the fuel cell apparatus. Micro-X-ray CT scans can capture the microscopic cracks on the MPL surface. Cracks on the MPL dictate the path that a liquid must take to penetrate the GDL structure. Results showed that the addition of the MPL results in higher saturation levels and a more uniform liquid distribution across the sample; an example is shown in Figure 23.

**Figure 24:**
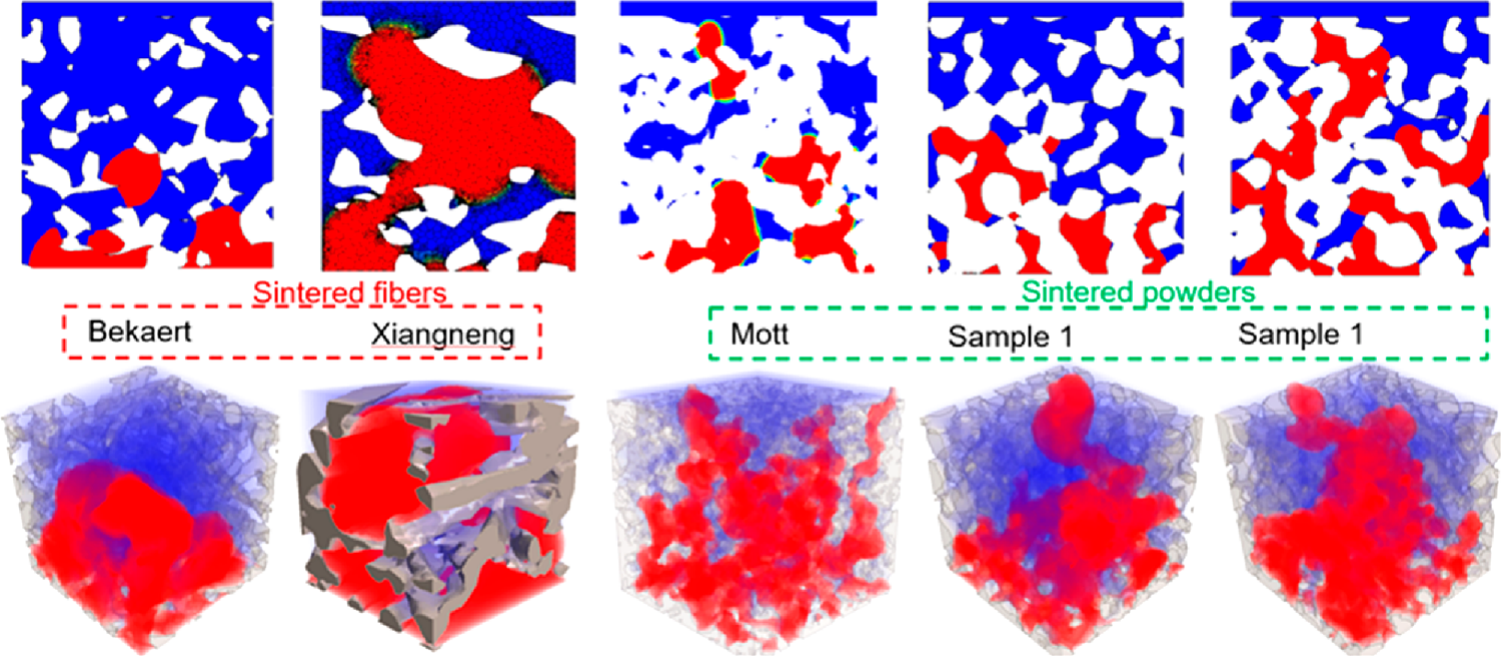
Predictions of oxygen transport pathway inside five samples of PTL using VoF method (Top: 2D cut plane at the middle of the sample; Bottom: 3D representation of bubble evolution). Adapted with permission from ref (252).

Sepe^252^ developed a numerical modeling technique to predict oxygen bubble evolution through saturated PTL. Previously, complex pore geometries were difficult to mesh, so the LBM was employed. LBM utilizes particle-based tracking to simulate liquid–solid interactions but requires time steps between 1 × 10^–10^ and 1 × 10^–8^ s. Now, better X-ray CT technology allows for more precise geometry reconstruction. Accompanied by an improved meshing process, the finite volume method can be used to simulate transport through porous media. This allows for larger time steps when running simulations. In this study, each PTL geometry is reconstructed using image processing techniques that take X-ray CT scans and generate 3D geometry. Simulations use the volume of fluid (VoF) method to predict two-phase flow through different PTL structures. Results showed that bubble formation will penetrate the largest pore volume available. Fibrous material will tend to have larger bubbles present inside the sample because the pore volume is larger. Sintered powder material has smaller bubbles but more breakthrough paths for bubble evolution. Growth, surface interaction, evolution, velocity, and size of oxygen bubbles through the microstructures of the PTL samples were observed. The results are summarized in Figure 24.

## 5 Summary and Future Directions

### 5.1 Summary of Findings for X-ray CT

X-ray CT is an imaging technique that can show the inner structure of the crucial electrochemical cell components. In this review, studies into the use of X-ray CT in in situ and operando studies for different electrochemical purposes have been presented. In all, catalyst synthesis, fuel cells, electrolyzers for water and CO_2_ reduction, and redox flow battery studies have been reviewed. Once the images have been captured on the beamline, the image analysis afterward gives numerical and visual data to characterize the cell being studied. Common to all these studies is the morphological information obtained in terms of layer porosity, various phase distributions such as gas or liquid, and catalyst distribution. X-ray CT provides feedback on component design, such as the morphology or wettability of particular layers. Specifically, X-ray CT is useful when rationally designed electrodes are needed. X-ray CT can precisely quantify gradients in porosity, pore-sizes, or wettability, and it can confirm their effectiveness on mass transport. Furthermore, degradation mechanisms for all the devices mentioned and their electrocatalysts can be explained with X-ray CT: loss of mass due to corrosion, change of wettability, or catalyst redistribution can all be visualized with X-ray CT. The results of these studies have contributed to helping improve the knowledge about device functionality, degradation due to use, and mass flow inside of the cells.

X-ray CT can provide morphological data to find crucial values for catalysts such as porosity, tortuosity, and catalyst surface area.^156,157,253^ Pyrolysis is a common way to synthesize catalysts, and X-ray CT can be used for in situ measurements of the morphological changes throughout pyrolysis. Micro-CT in situ testing showed that porosity increased with temperature. Similar studies have been applied to reprolysis where only minor morphological changes were seen. Nano-CT is also applicable to catalyst studies. Ex situ nano-CT was used on Fe–N–C to find that most of the metal oxides were on the surface, and in situ nano-CT was able to track specific particles and found that the particles became smaller at the beginning of pyrolysis.

Both micro- and nano-CT can be used for fuel cell studies as well, but nano-CT is not commonly used for polymer membrane fuel cells. These techniques can help to study degradation, reactivity, and mass transport inside fuel cells. In this review, the studies that were focused on were studies into water management and material degradation using micro-CT. By introducing water vapor to MPLs and using X-ray CT to image the cell, it was found that electrode morphology and the wettability of the interfaces control water distribution inside the catalyst layer.^171^ X-ray radiography can be used along with X-ray CT to help visualize the water better. Another test of adding PTFE to the catalyst ink helped to prevent flooding and improved performance. For degradation studies, X-ray CT combined with AST showed that more carbon is lost from areas under the land part of the flow field than from under the channel areas.^182^ However, in a study about crack formation, it was found that the membrane degrades more in the channel areas than the land areas.^115^

X-ray CT allows 3D visualization inside electrolyzers which is advantageous to study the catalyst utilization and morphology of the PTLs.^197,198^ Oxygen transport through different types of PTLs can be characterized, which helps optimize the design of PTLs. It was found that the presence of an MPL allows for more interfacial contact with the catalyst layer, and refining the PTL structure can help reduce mass transport overpotentials. The information gathered here can also be used to help with CO_2_RR electrolyzers. CO_2_ reduction relies heavily on GDEs, and the issues of flooding and fluid transport can be investigated by using X-ray CT. Insights from PEMWE X-ray CT studies along with other X-ray imaging techniques can help influence CO_2_RR electrolyzer design.

Vanadium redox flow batteries utilize a porous carbon felt electrode, which presents a couple ways that X-ray CT can be used to provide data. Morphological data about the electrode structure and performance and studies into electrolyte flow are the main focuses here. X-ray CT can be used with X-ray radiography here as well to help with the catalyst visualization. Porous electrodes with and without bismuth were imaged in operando tests which showed that the use of a heterogeneous catalyst can help improve the overall performance.^33^ Electrolyte flow depends on a couple factors. Flow field design has an effect, and a study found that a serpentine design leads to higher electrode saturation with continuous electrolyte flow.^34^ Electrode compression also affects saturation. It was found that saturation increases with compression up to 97% saturation around 50% electrode compression.^35^

Mathematical modeling using data from X-ray CT imaging can allow for different porous structures to be investigated. CFD can be applied to reconstructed geometries of samples to simulate the fluid flow dynamics and transport phenomena that occur inside the porous layers of the electrochemical cells being investigated.^111,249−251^ In this way, for example, the effects of MPLs and different porous structures can be compared using models to gather the overall cell performance in a quicker manner than a full experiment for each type of porous layer.

### 5.2 Future Methods

In electrocatalysis, X-ray CT is especially useful when catalysts are integrated into real devices and operated under realistic conditions of temperature, pressure, relative humidity, and applied potential or current density. Then X-ray CT can detect morphology changes and new phase formation (gas, liquid) and diagnose material degradation. Both steady-state and transient techniques are useful, as the steady-state method allows for higher spatial resolution compared to dynamic imaging, while transient simulations can detect dynamics of new phase formation. Currently, dynamic X-ray CT scans are possible due to advances in detectors, optics, and high X-ray flux. However, challenges exist with operando systems as these fuel cells or electrolyzers have tubing for reactants/products and electric leads that must rotate with the cell. For subsecond scans, the cell accelerates fast and will complete several complete rotations before coming to a stop. This means that all of the wires and tubing must rotate at the same speed. Designing slip-rings at the beamlines for tubing and electric leads is critical to accommodate cell rotations. For example, TOMCAT^254^ at PSI has such a slip-ring to accommodate subsecond scans and currently is the only beamline that produced subsecond scans on operando electrochemical devices.

For nano X-ray CT, challenges exist with operando cell development for electrocatalysis studies. Nano X-ray CT beamlines have small spot X-rays with high flux; therefore, beam damage becomes significant. Furthermore, the beamlines that use zone plates are restricted to an energy range up to about 12 keV, as the manufacturing of zone plates beyond this range is challenging. This region is characterized by tender X-rays to low hard X-rays. As discussed earlier, beamlines with K–B mirrors are not limited to low-energy ranges and can alleviate the issue of beam damage. Alternative optical configurations exist too, such as compound refractive lenses that are designed for hard X-ray imaging (>15 keV)^255^ for full-field imaging.

Many materials will attenuate X-rays in this low-energy range, and therefore, operando cell design is limited to soft and thin materials, such as Kapton film and thin PEEK materials. Furthermore, because of high resolution, the field of view is only on the order of 40–100 μm. Most of the operando cells will be larger than the field of view with significant portions of hardware falling outside of the imaging FOV. This will introduce reconstruction artifacts. With 30 nm resolution, the hardware must be mounted properly and be stable. When gaseous reactants are introduced or products are produced, it is inevitable that components will swell or move on a nanometer scale. Therefore, an additional challenge exists for imaging at the nanoscale as electrocatalysis systems are inherently dynamic during operation. Many of the electrochemical systems rely on soft materials, such as carbon-based materials, oxides, or polymers. To image soft materials, a phase-contrast imaging mode is needed. Typically, a phase-ring is placed between the stage and detector, and images are collected in phase and not absorption mode. The challenge of phase-contrast imaging is that it is slower than absorption mode, and complex algorithms are needed to retrieve the phase; otherwise, phase artifacts will be present in reconstructed images. Many beamlines opt to not have phase-contrast nano-X-ray CT imaging and prefer to focus on absorption imaging. Beam damage is still significant at these low energies; therefore, a system with any polymer or water will see damage. Some of these challenges can be alleviated by transitioning to different optical configurations using K–B mirrors that allow nano-X-ray CT imaging at higher energies. ESRF Beamline ID16A is an example that uses K–B mirrors.^256^ The challenge with K–B mirrors is that the beamline must be extremely stable both vibrationally and thermally.

Operando nano-CT of PEFCs is critical as it can enable visualization of water distribution in the catalyst layer, and it can also show catalyst porosity changes and wettability changes after degradation experiments. If beam damage is not an issue, one can visualize these phenomena during operation. Phase-contrast is needed to visualize carbon agglomerates, whereas absorption is needed to observe catalysts that have a higher X-ray attenuation coefficient (such as metals).

In the future, especially for the electrocatalysis field, we will see the development of X-ray CT techniques combined with XAS or XRF, where chemical information can be extracted and overlaid onto 3D morphological domains obtained with X-ray CT. For example, nano-X-ray CT beamlines already have the capability of 3D XANES. This requires imaging to be fast, as for each energy one must have a 3D scan. Then the limitation shifts away from spectroscopic imaging to data processing. Each sample will have about 100 scans, with each corresponding to a discrete energy level. These scans must be reconstructed and processed for the chemical information. Having an automatic algorithm and ML methods to do this will be much needed. Combined X-ray CT and XAS or XRF will enable either elemental mapping (XRF) or oxidation state (XAS) of the catalyst. For example, during degradation studies, catalyst loss can be observed with X-ray CT, but the loss can be quantified with XRF, which allows for precise loading detection. During PEMWE operation, IrOx undergoes various oxidation states during current cycling (Ir–III to Ir–IV, for example), and its initial performance can be correlated with the XAS oxidation state; whereas upon degradation, IrOx can change its oxidation state, and its morphology will change too. Tracking both the oxidation state of the catalyst and the morphology of the catalyst layer is critical to design low-loading IrOx PEMWE cells.

In terms of modeling X-ray CT data, scale-bridging models that can incorporate data from micro- and macroscales will be used more often to predict reaction kinetics and transport phenomena across a multiscale range becauseelectrochemical devices have porous layers that range from nanoscale to macroscale in pore-sizes. XAS, XRF, and other additional probes will be used to validate the models’ capabilities to predict chemical and electrochemical information. Transient simulations on geometries generated by X-ray CT will be needed to simulate transient physics within the devices, such as bubble nucleation, growth, transport, and coalescence during electrolysis or water removal from the catalyst layer within fuel cells.
